# Assessing comparative importance of DNA sequence and epigenetic modifications on gene expression using a deep convolutional neural network

**DOI:** 10.1016/j.csbj.2022.07.014

**Published:** 2022-07-13

**Authors:** Shang Gao, Jalees Rehman, Yang Dai

**Affiliations:** a Department of Biomedical Engineering, University of Illinois at Chicago, Chicago, IL, USA; b Department of Medicine, Division of Cardiology, University of Illinois at Chicago, Chicago, IL, USA; c Department of Biochemistry and Molecular Genetics, University of Illinois at Chicago, Chicago, IL, USA; d University of Illinois Cancer Center, Chicago, IL, USA

**Keywords:** Epigenetic modifications, DNA sequence, Transcription factors, Multi-omics, Deep convolutional neural network, Deep learning, Prediction of gene expression, Integrated gradients

## Abstract

Gene expression is regulated at both transcriptional and post-transcriptional levels. DNA sequence and epigenetic modifications are key factors which regulate gene transcription. Understanding their complex interactions and their respective contributions to gene expression regulation remains a challenge in biological studies. We have developed iSEGnet, a framework of deep convolutional neural network to predict mRNA abundance using the information on DNA sequences as well as epigenetic modifications within genes and their *cis*-regulatory regions. We demonstrate that our framework outperforms other machine learning models in terms of predicting mRNA abundance using transcriptional and epigenetic profiles from six distinct cell lines/types chosen from the ENCODE. The analysis from the learned models also reveals that specific regions around promotors and transcription termination sites are most important for gene expression regulation. Using the method of Integrated Gradients, we identify narrow segments in these regions which are most likely to impact gene expression for a specific epigenetic modification. We further show that these identified segments are enriched in known active regulatory regions by comparing the transcription factor binding sites obtained via ChIP-seq. Moreover, we demonstrate how iSEGnet can uncover potential transcription factors that have regulatory functions in cancer using two cancer multi-omics data.

## 1 Introduction

Precise regulation of gene expression is an essential biological process for all cells because it allows for fine-tuned coordination of complex transcriptional programs. Multiple regulatory mechanisms work in concert to regulate transcription of individual genes. First, chromatin accessibility is required for the binding of transcription factors to initiate gene expression; such chromatin accessibility can be assessed by DNase I hypersensitivity [1]. Histone modifications robustly dictate chromatin structure and thus are important regulators of gene expression. For example, H3K4 trimethylation (H3K4me3) is commonly associated with the activation of transcription of genes in the proximity of the modification through chromatin remodeling by the NURF complex [2]. Second, DNA methylation is another type of epigenetic modification that regulates gene expression; it is associated with closed chromatin and is thought to repel DNA Polymerase II or transcription factors from binding to loci such as promoters or enhancers [3]. Lastly, genetic variants in gene regulatory regions can also affect all the above-mentioned mechanisms by changing the interactions between the DNA sequence and regulatory modifications or proteins [4]. These diverse aspects of gene regulation have been interrogated using next-generation sequencing technologies such as RNA-seq, ChIP-seq, and whole-genome bisulfite sequencing (WGBS) [5], [6]. Joint modeling of these data within a quantitative framework has the potential to shed light on their relative importance, to elucidate mechanistic underpinnings, and to uncover new modes of gene regulation [7], [8].

Several computational tools have been developed to identify the relationship between histone modifications and gene expression [9]. Multiple machine learning models are being used to predict gene expression from epigenetic profiles, including linear regression [9], support vector machine (SVM) [10], and random forest combined with regression [11]. These methods take a “binning” approach to divide a large region surrounding the gene transcription start site (TSS) and transcription termination site (TTS) into consecutive smaller bins to represent the epigenetic signals. However, histone modification signals can span over an increasingly long range [12] that requires representation by multiple bins. Thus, the above machine learning methods can not completely capture the relationship of the neighboring bins, because the relationship of the bins is not explicitly explored in these machine learning models.

Recent years have witnessed the rapid growth of applications that leverage deep learning in systems genomics to learn complex non-linear relationships from input data for prediction tasks [13]. Convolutional neural networks (CNNs) have been used successfully in multiple studies, such as predicting the transcription factor binding sites [14] and classifying cell types in single-cell RNA-seq data [15], in part due to the CNNs’ capacity to capture both local and global representations, which are important for accurate predictions. Several CNN models are specifically designed for gene expression prediction using various inputs. Some models have achieved state-of-the-art performance using DNA sequences in the human and mouse genomes [16], [17], [18], [19], [20]. These models rely on population data or aggregated data from multiple mRNA expression datasets, limiting their application to cases where multi-omics profiles are generated only for a few biological replicates. Other models, such as DeepChrom and DeepDiff, use histone modification signals to predict gene expression level (i.e., low or high) and differentially expressed genes [12], [21]. Other methods that predict gene expression only consider open chromatin regions (DNase I hypersensitive sites) and DNA methylation signals, without incorporating DNA sequences of genes into the models [12], [22]. Overall, this emphasizes the need for a model which integrates the effects of both DNA sequence and epigenetic modifications, and identify their interactions with on gene expression regulation.

We present a novel framework herein to assess the comparative importance of DNA Sequence and Epigenetic modifications on Gene expression regulation using a deep convolutional neural network (iSEGnet). By incorporating an analytical approach known as Integrated Gradients (IG) [23] in a trained neural network model, iSEGnet further computes an attribution score associated with a specific epigenetic modification for each position in the input DNA region. This attribution score indicates the relative magnitude of the potential impact of the epigenetic modification on gene expression at that specific position. We demonstrate that the iSEGnet models outperform other machine learning models for predicting mRNA expression levels in terms of Transcripts Per Kilobase Million (TPM) using data of six different cell lines/types from the ENCODE project [6]. The attribution analysis also reveals positions in the *cis*-regulatory regions that are important for predicting gene expression. We further show that these regions are active regulatory regions by analyzing them for ChIP-seq derived transcription factor binding sites. Moreover, we apply iSEGnet to data obtained from two cancer datasets to identify putative regulatory transcription factors specific to the disease conditions.

## 2 Materials & methods

### 2.1 Datasets and preprocessing

To train an iSEGnet model, three types of data, i.e., DNA sequences of genes, epigenetic modification signals, and observed mRNA levels (gene expression), are required. To train and evaluate iSEGnet, we used epigenetic modification and gene expression data from six different cell lines/types provided in the ENCODE project [6]; namely, A549 (adenocarcinomic human alveolar basal epithelial cells), HepG2 (Human liver carcinoma cells), K562 (human immortalised myelogenous leukemia), large intestine (human intestine tissue male embryo, 108 days), pancreas (human pancreas tissue make adult, 34 years), and small intestine (human small intestine tissue make child, 3 years). The epigenetic data includes DNase-seq (indicative of open chromatin and comparable to ATAC-seq data), ChIP-seq of five histone modifications (H3K4me1, H3K4me3, H3K9me3, H3K27me3, H3K36me3), and WGBS DNA methylation profiles. The DNA sequences of genes were obtained from the hg38 reference genome in NCBI.

We additionally acquired datasets of esophageal tumor [24] and breast cancer multi-omics [25] studies from Gene Expression Omnibus (GEO) (accession numbers: GSE149612 and GSE118716). The esophageal tumor study provides the WGBS DNA methylation and gene expression profiles in both tumor and normal tissues from 9 patients. The breast cancer study provides histone modifications (H3K4me1 and H3K4me3), WGBS DNA methylation, and gene expression profiles in one drug-sensitive breast cancer cell line (MCF7, endocrine-sensitive) and one drug-resistant cell line (TAMR, endocrine-resistant derivatives tamoxifen-resistant). These two cancer-related datasets were used as case studies for the application of iSEGnet. The details about cell lines and cell types are described in Supplement Table 1.

The preprocessed histone modification and DNase-seq data were downloaded. They provide the signal p-values in bigWig format processed with Bowtie2 and MACS [5]. A p-value indicates the significance of a signal in that region compared to the control input. The whole genome was divided into 20 bp length regions. The p-value was assigned to each region as follows. First, we assigned the p-value to each site within that region. Then, we transformed the p-value by -log_10_ and scaled it to [0,1].

For DNA methylation data, we downloaded the preprocessed raw signal data in bigWig format. Each value ranges from [0, 100], indicating the percentage of methylation on that site. We assigned all other sites with 0, which means unmethylated, and then scaled the data into [0,1].

For DNA sequence data, we used one-hot encoding to transform DNA sequences into a binary-valued matrix. An input sequence of length L was represented by a L× 4 matrix, where 4 is the number of nucleotides (A, C, G, and T). The selection of L will be discussed in Section 3.1.

For gene expression data, we downloaded the normalized count tables and removed the genes with zero expression.

### 2.2 iSEGnet architecture

**Fig. 1:**
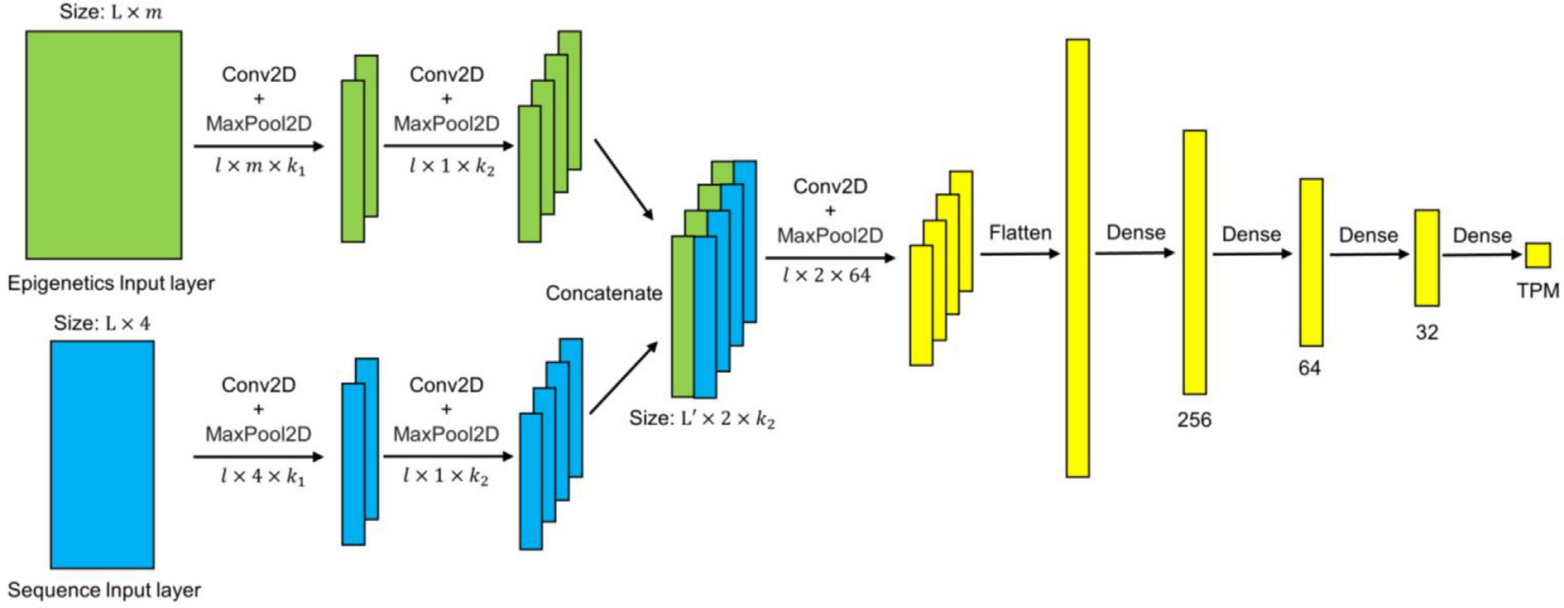
iSEGnet architecture. iSEGnet is a deep convolutional neural network with two-modality inputs. The network predicts gene expression by epigenetic modifications and DNA sequence of the regions around the transcription starting site and transcription termination site of a gene. l is the number of rows of a kernel. m is the number of epigenetics modification types in input. $k_{1}$ and $k_{2}$ are the numbers of kernels in the first and second convolutional layers, respectively.

iSEGnet is a deep convolutional neural network framework in which each gene is considered an input sample. As shown in Fig. 1, iSEGnet has two key input sources, i.e., the epigenetic modification signals and the DNA sequences of the gene regulatory regions. As the output, it predicts the mRNA abundance level (TPM) of the gene. Therefore, to train the model, the RNA-seq gene expression measured under the same condition is required. iSEGnet consists of several convolution layers. The convolutional layers contain multiple convolution kernels, each of which extracts features from a single perspective. The first convolutional layer extracts low-level features from the original data. The next convolutional layer pulls out high-level features from the low-level features, and its convolution kernel size gradually decreases. The rectified linear unit (ReLU) is used as an activation function at each node of the network, i.e.,

$$\mathrm{ReLU}=\left\{\begin{array}{cc} x & x>0\\ 0 & x\leq 0 \end{array}\right)$$

We also used dropout for model regularization to avoid overfitting. To train a model, first, the input corresponding to the epigenetic data is fed into a convolutional layer with l×m × $k_{1}$as the kernel size. The value of m is determined for each dataset based on the number of available epigenetic modifications in a dataset. The input corresponding to the DNA-sequence is fed into a convolutional layer withl × 4 × $k_{1}$as the kernel size. Next, a max-pooling layer follows the convolutional layer to further preserve the features with the highest scores in a region. Then, there is another convolutional layer with kernel sizel × 1 × $k_{2}$and a max-pooling layer, where the two input components (the epigenetic modifications and the DNA sequence) of the same size (L′ × 1 × $k_{2}$) are concatenated by columns and used as input to the last convolutional (kernel size: l×2 × 64) and max-pooling layer. Next, the learned region representation is fed into four fully connected layers. The final output of the iSEGnet framework is the mRNA abundance level in TPM count of the input gene. TensorFlow 2.0 [26], [27] was used to train iSEGnet. $l,k_{1},k_{2}$ are Hyper-parameters that will be tunned.

### 2.3 Model training and testing

iSEGnet was trained using the ADAM optimizer [28] with the loss function.

$$\mathrm{MSE}=\frac{1}{N}\sum_{i=1}^{N}{\left(y_{i}-y_{i}^{\prime }\right)}^{2}+\lambda \sum {\left|\left|W\right|\right|}_{2}$$

Here, N is the number of genes in training data. The first term is the mean squared error (MSE) between the observed gene expression $y_{i}$ and the predicted expression $y_{i}$. The second term is the L2 regularization with a penalty parameter λ.

The data for each cell type were divided into three parts: training (60 %), validation (20 %), and testing (20 %). The network was fine-tuned for 200 epochs using a batch size of 100. Batch normalization layers were added to the network to increase stability. Early stopping was applied by checking the model performance on the validation set at every epoch during the training to avoid overfitting. The prediction results on the test sets were used to report model performance.

Hyper-parameter tuning was performed to determine the optimal set of hyper-parameters (number of layers, number of kernels, kernel size, the L2 regularization parameter λ, and dropout rate). We allowed for two convolutional layers in each of the convolutional networks. The number of kernels varied from the combination of 16, 32, 64, and 128 with different sizes: 20 × 7, 50 × 7, and 100 × 7. The value of *λ* was selected from 5 different values: 0.0001, 0.01, 0.1, and 1.0. The dropout rate was selected from 0.1, 0.3, and 0.5. The hyper-parameters and the corresponding model performance using the evaluation criteria described in Section 2.4 are shown in Supplement Fig. S1. Our analysis indicates that the models with the hyperparameters (64 nodes for the first and 128 nodes for the second convolutional layers, kernel size of 20x7, dropout rate of 0.5, and *λ* value of 0.0001) generated the best performance. Therefore, we chose these hyperparameters for the rest of our study.

We also evalued another architecture as follows. Instead of using a convolutional layer to extract information from the concatenation layer, we used the fully connected layers right after the concatenation layer. The evaluation on the test sets indicated a better performance of iSEGnet (Supplement Fig. S2).

### 2.4 Evaluation criteria

The best models were chosen according to the coefficient of determination (R^2^) computed on the test set of a cell type, i.e., $R^{2}=1-SSResidual/SSTotal$, where SSResidual is the sum of residual squares of predictions and SSTotal is the total sum of squares from the observations. We also used Pearson’s correlation coefficient between the observed and predicted expression as an additional measurement to evaluate the models. These criteria were considered because the iSEGnet predictions are gene expression levels.

### 2.5 Comparision with other gene expression prediction methods

We used the Python library (scikit-learn) [29], [30] to compare with other machine learning models, including random forest and support vector machines. We used *sklearn.ensemble.RandomForestRegressor* with 100 and 200 trees for random forest, and *sklearn.svm.SVR* (rbf and linear kernel) and *sklearn.svm.NuSVR* (rbf kernel) for support vector machines. We conducted the grid search for hyperparameter tuning for random forest and support vector machines using 5-fold cross-validation and reported the mean R^2^ values for each combination of hyperparameters (Supplement Fig. S3, Supplement Table 2, and Supplement Table 3). These models were trained and evaluated on the same training and testing data used for the iSEGnet models.

### 2.6 Feature attribution identification

In order to identify the most relevant modifications and locations for predicting gene expression, we used the method of Integrated Gradients (IG) [23] to compute the attribution of every site on input sequence regions. IG explains predictions from a differentiable function F defined on a feature space X and F is obtained from the trained deep learning models. The per-feature attributions for a prediction are defined relative to a reference point x′∈X and its prediction F(x′). For an observation x∈X, IG obtains an attribution vector attr(x) by integrating the gradients of F with respect to the feature space along a path γ:[0,1]→X that starts at x′ and ends at x, i.e., γ(0)=x′ and γ(1)=x.

Sundararajan et al. focus on a special case where the path γ is chosen to take the straight-line path from x′ to x. Parameterized by α∈[0,1], the path is γ(α)=x′+α(x-x′) so that the attribution for the j th feature is defined as.

$$\mathrm{att}r_{j}\left(x\right):=\left(x_{j}-x_{j}^{\prime }\right)\times \int_{0}^{1}\alpha \partial F\left(x^{\prime }+\alpha \times \left(x-x^{\prime }\right)\right)\partial x_{j}d\alpha$$

The IG analysis was performed using function *alibi.explainers.IntegratedGradients* [31] with *n_steps = 200*.

### 2.7 Transcription factor ChIP-seq data for validation

To assess whether high attribution regions were related to known transcription factor binding regions, we used the MYC transcription factor ChIP-seq data in the lung cancer cell line A549 as an example. The ChIP-seq data were downloaded from the ENCODE project (ENCSR000DYC), and the p-values of the peaks were transformed by -log10 as the MYC binding signal on each site.

### 2.8 KEGG enrichment analysis

Enrichr [32], [33], [34] was used to identify signature KEGG pathways [35] of the transcription factors identified by high attributions in iSEGnet. Fisher’s exact test was used to perform the enrichment testing. The Benjamini-Hochberg method was applied for the multi-test correction, and 0.05 was set as the significant threshold for adjusted p-values.

## 3 Results

### 3.1 Identify the optimal combination of input regions for gene regulation

**Fig. 2:**
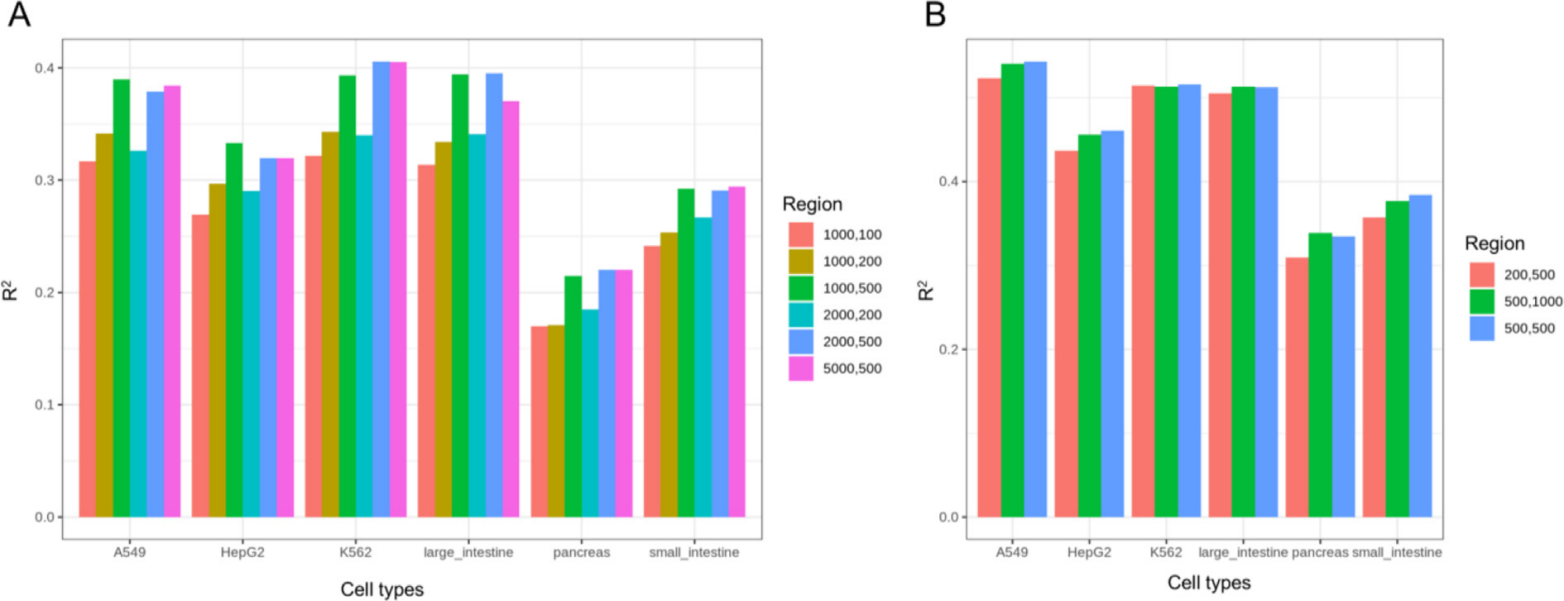
The performance of iSEGnet with distinct input regulatory regions. (A) The R^2^ values of the iSEGnet models on the testing datasets with different regions around TSS as input for the six cell lines/types. The region [-1000 bp, +500 bp] around TSS has the best performance across datasets. (B) The R^2^ values of the iSEGnet models on testing datasets with [-1000 bp, +500 bp] around TSS combined with different regions around transcription termination sites as inputs for the six cell lines/types. The region [-1000 bp, +500 bp] around TSS plus [-500 bp, +500 bp] around TTS has the best performance across datasets. This region will be the final input for iSEGnet.

Four key *cis*-regulatory regions, i.e., promoter, 5′-UTR, 3′-UTR, and terminator, were evaluated as inputs for iSEGnet. It is not fully understood how these regions differentially impact gene expression in the setting of distinct epigenetic modifications. The respective lengths of these regulatory regions for each gene are also not well-defined. To identify the regulatory regions which best predict gene expression, we created combinations of these regions with different lengths as input. We trained models separately with these different regulatory regions to find the combination with the highest performance evaluated in the test sets (details see Methods). First, we only used promoters and 5′-UTRs as inputs to the model. We first assessed different lengths of promoters and 5′-UTR. Among various combinations, 1000 bp upstream of TSS (and 500 bp downstream of TSS ([-1000 bp, +500 bp] around TSS) were the regions with the best coefficient of determination (R^2^) values across all six lines/cell types (Fig. 2 A). Then we fixed the promoters and 5′-UTR and combined them with different lengths of 3′-UTR and terminator regions. We found that model performance increases 0.10–0.15 in R^2^ values in all cell types (Fig. 2 B). Importantly, this analysis highlights that the regions around the TTS also impact gene expression. The performance of models was most consistent when we set 3′-UTR as 500 bp upstream of TTSs. Therefore, we chose regions of [-1000 bp, +500 bp] around TSSs and [-500 bp, +500 bp] around TTSs as the input to obtain the model for subsequent analysis. As a negative control of the model, we generated a random dataset by shuffling gene expression randomly and trained the model on the data set. The R^2^ values of the models on the test sets are close to 0 (e.g., R^2^ value was −0.02004 for K562 and −0.001861 for A549), demonstrating the prediction of iSEGnet model is not due to randomness.

### 3.2 The iSEGnet models outperformed other machine learning models

**Fig. 3:**
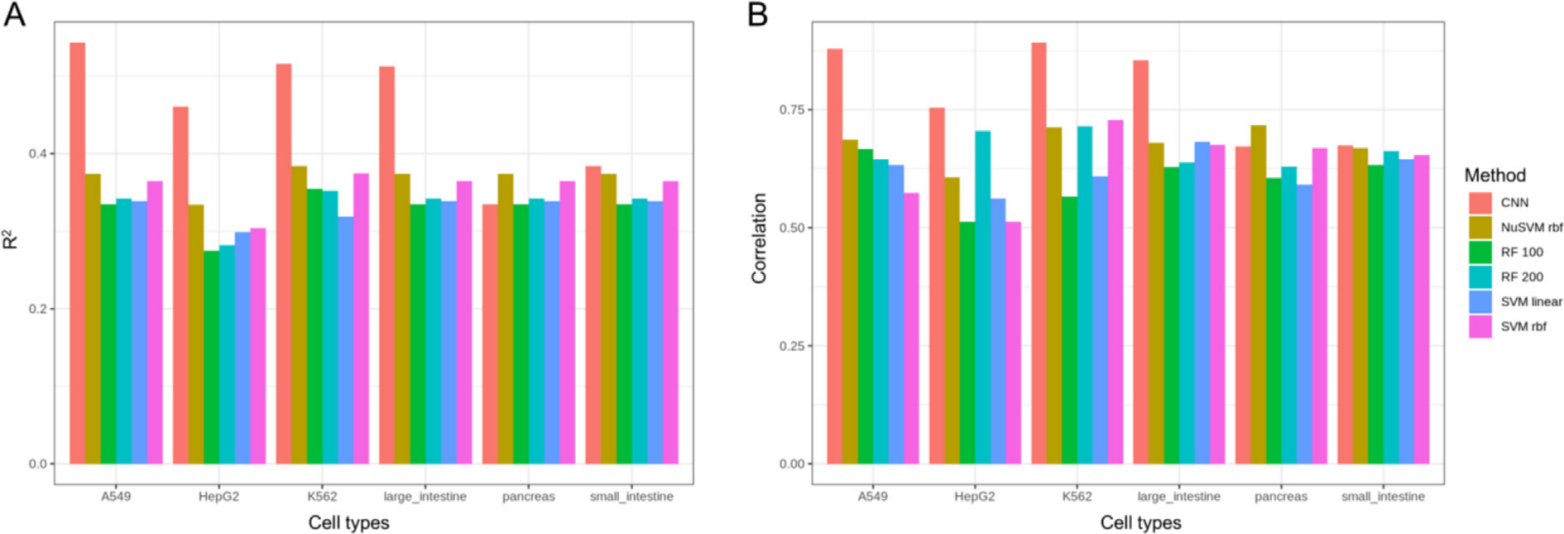
The performance of iSEGnet compared with other machine learning models. (A) The R^2^ of prediction with different models on the testing datasets. (B) Pearson’s correlations between the prediction and overserved expression with different models on the testing datasets.

Next, we compared iSEGnet to other widely used machine learning methods, including random forest and support vector machines. iSEGnet outperformed other machine learning models in three cell lines (A549, HepG2, and K562) with increases of 0.15–0.30 in R^2^ values and Pearson’s correlations on the tested datasets (Fig. 3 A, B, Supplement Fig. S4). In primary cells, iSEGnet also outperformed these methods for the large intestine dataset and performed comparably for the other two cell types (pancreas and small intestine). We next compared with DeepChrom, a classification model to predict binary gene expression levels with histone modification signals. The developers used a binary approach, labeling the expressed genes as either “high expression genes” or “low expression genes”. The median of all gene expression values was used to separate the genes into these two groups. As our model predicts normalized gene expression values instead of using the binary approach of DeepChrom, the settings of these two models are fundamentally different. Thus, we modified iSEGnet slightly in order to perform the comparison. First, we labeled the genes as “high expression genes” or “low expression genes”, analogous to the DeepChrom approach. Then we used the binary cross-entropy as the loss function and kept all other aspects unchanged. The performance of DeepChrom and the classification version of iSGEnet is shown in Supplement Fig. S5. Our model has a higher accuracy for all six cell lines and cell types compared to DeepChrom.

### 3.3 The impact of different epigenetic profiles on gene expression prediction

**Fig. 4:**
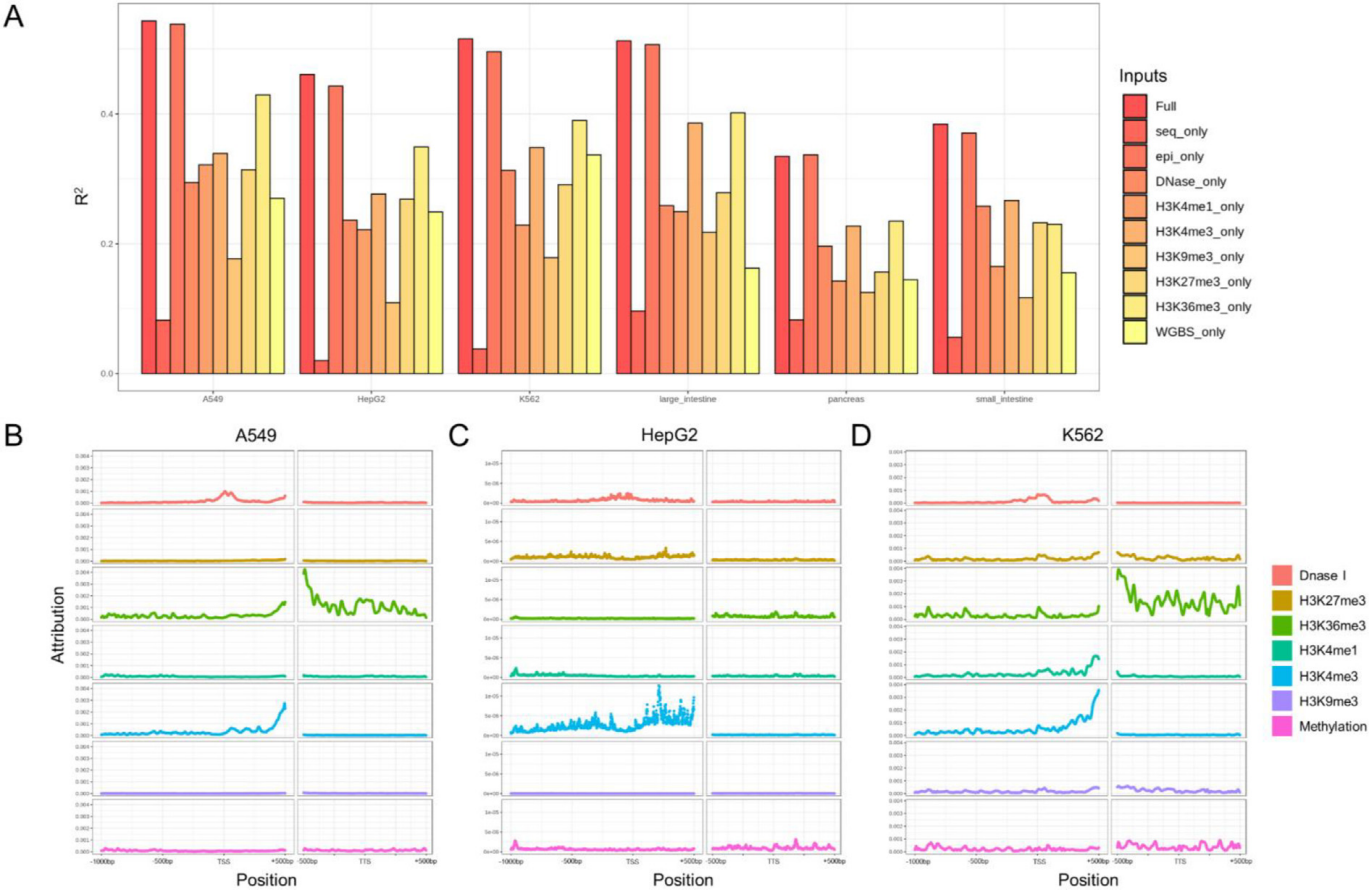
The importance of epigenetic modification on gene expression by dropout analysis and integrated gradient. (A) The R^2^ values of iSEGnet with different epigenetics modification data as input. (B), (C) and (D) The mean predicted site-specific attributions for each epigenetic modification on gene expression across all genes in cell lines A549, H3pG2, and K562, respectively.

Next, we investigated which epigenetic modification most impacts gene expression. Our strategy was to train the model using only one type of epigenetic modification to evaluate the performance of the input signal. In all cell types, we found that epigenetic modifications contribute significantly more to model accuracy than DNA sequences. Moreover, we observed distinct patterns of the importance of epigenetic modifications in different cell types (Fig. 4 A). This approach allowed us to assess the contributions of individual epigenetic modifications, as well as the DNA sequence to the overall performance. For all cell types, the models using only DNA sequence as input had the lowest prediction performance (paired *t*-test, p-value < 0.05) for RNA expression (R^2^ value < 0.1). When only one type of epigenetic modification was used as the input, the performance of the models varied for different cell types. In general, H3K36me3 and H3K4me3 were the most important epigenetic modifications for predicting gene expression [12]. Notably, the model integrating all epigenetic modifications and the DNA sequence showed the best performance, thus underscoring the need for a comprehensive and integrated approach when predicting RNA expression levels.

To discern the influence of epigenetic modifications, we compared the predicted gene expression in A549 from the model with epigenetics data only to the predicted gene expression from the model with both epigenetics data as well as DNA sequence. We selected the top 10 % of genes which showed minimal differences between these two models for the predicted expression levels. The predicted expressions of these genes were therefore less impacted by DNA sequence. We found that these genes had higher DNase ChIP-seq signals around TSS regions and lower DNA methylation levels on TSSs and 5′-UTRs compared to the rest of the genes (Kolmogorov–Smirnov test, p-value < 0.05) (Supplement Fig. S6). Similarly, we analyzed models trained with sequence data only and trained with both epigenetics data and DNA sequence. We identified motifs enriched on the genes that have little change in the predicted expression level compared to other genes (Supplement Table 4). These analyses indicate that iSEGnet may shed light on subsets of genes that are more likely to be regulated by DNA sequences or by epigenetic modifications, facilitating the design of additional hypotheses.

### 3.4 Attribution of an epigenetic modification at a given site in the regulatory region

After identifying the relative importance of distinct epigenetic modifications on gene expression, we proceeded to detect the DNA sequence regions with the highest regulatory attributions for gene expression. Using the IG method on the trained models from all six cell lines/types (Fig. 4 B,C,D; Supplement Fig. S7), we computed the attribution of each epigenetic modification occurring at each DNA sequence site. From the mean predicted site-specific attribution for each epigenetic modification on gene expression across all genes in cell line A549, we observed that DNase I signals (“open chromatin”) on the region around TSS were very important for predicting gene expression (Fig. 4 B). This result is consistent with the prior biological knowledge that the DNase I signals indicate the accessibility of the chromatin to other factors which interact with the DNA to regulate gene transcription. Other histone modifications, such as H3K4me3 and H3K36me3, significantly impacted gene expression when the modifications occurred in 5’-UTRs. On the other hand, the epigenetic modifications of H3K36me3 were most important in TTS regions. Similar patterns were observed for cell line K562 (Fig. 4 C). The mean site-specific attribution in the HepG2 cell line showed patterns distinct from those seen in A549 and K562 cell lines (Fig. 4 D). For example, the H3K36me3 modification has much lower attributions around TTS regions in HepG2. These analyses indicate that the relative region-specific importance of certain epigenetic modifications are cell type-dependent.

Next, we used MYC as an example to show the integrated gradient attributions for an individual gene in the A549 lung cancer cell line. MYC is a well-known oncogene; it plays an important role in cell cycle progression and apoptosis [36]. For the attribution of the DNase I signal (“open chromatin”), we observed two regions with high attribution values. One was 500 bp upstream of TSS, and the other at the nearby TSS (Supplement Fig. S8 A). For the H3K4me3 signals, a high attribution region on 5’-UTR was observed, indicating that gene expression may be highly influenced by the H3K4me3 presence region (Supplement Fig. S8 B). Moreover, compared to the experimentally observed signals, there were shifts in the location and strength of the high attribution regions (Supplement Fig. S8 C, D). These results demonstrated that the attribution computed from our models is not simply a reflection of input data; but instead shows the relative importance of each region under a specific epigenetic modification for determining gene expression.

### 3.5 The high attribution regions are related to transcription factor binding

After identifying the attribution of each epigenetic modification on each site, we examined the possible biological functions of the regions with high attribution scores. We hypothesized that these regions may be related to transcription factor binding activities that regulate gene expression, thus explaining why changes in epigenetic modifications of these areas could have a significant impact on mRNA levels.

**Fig. 5:**
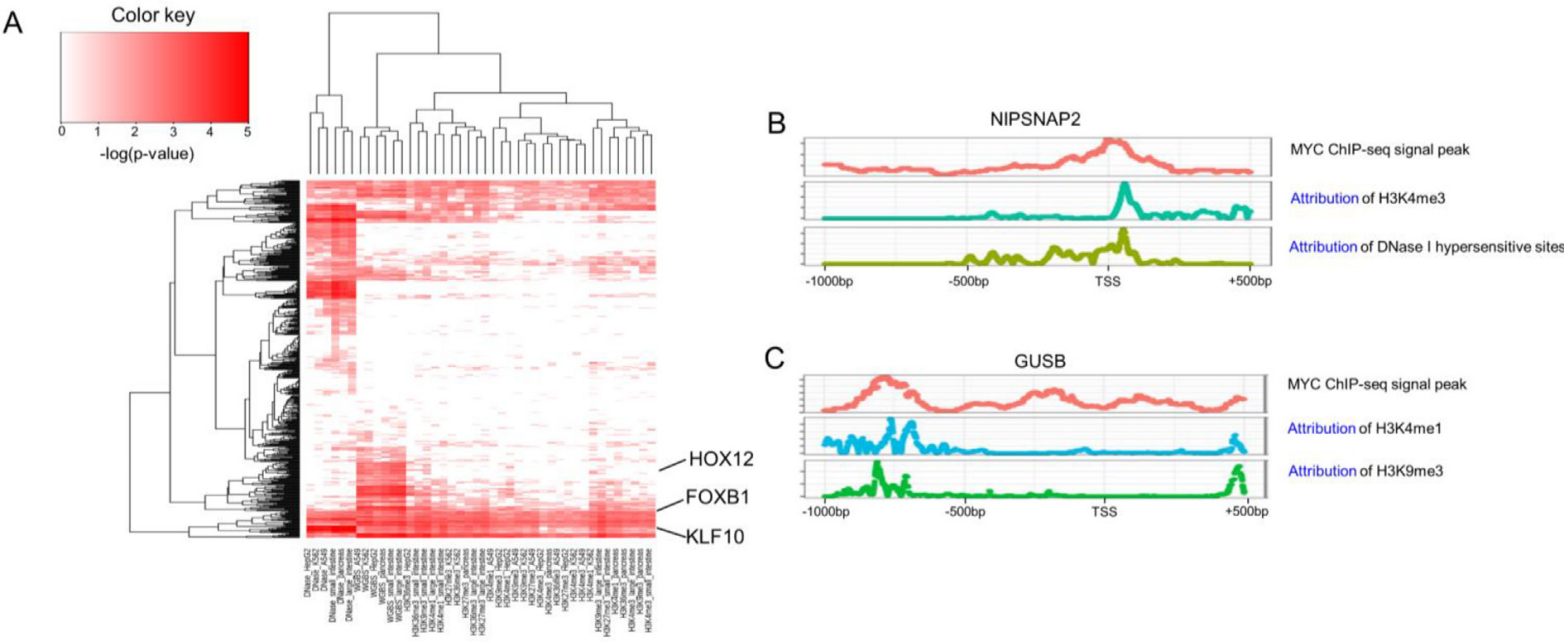
The transcription factor binding motifs are enriched on the high attribution regions identified by iSEGnet. (A) The enrichment pattern of transcription factor binding motifs on high attribution regions across all cell types and epigenetics modifications. (B) and (C) The MYC ChIP-seq signals and epigenetic modification attributions on the NIPSNAP2 and GUSB promoter regions, respectively.

To test this hypothesis, we first extracted the sequence of [-50 bp, +50 bp] around the highest attribution site of each gene for each epigenetic modification. Then, we used the method AME [37] from the MEME suite [38] to identify the enriched transcription factors binding motifs in the regions for each epigenetic modification. The transcription factor binding motifs were acquired from the JASPAR database [39]. From this analysis, we identified the transcription factors that might bind to these high attribution regions. We conducted the same analysis for every cell type. As observed in Fig. 5 A, the heatmap showed the enrichment pattern of transcription factor binding motifs on high attribution regions across all cell types and epigenetics modifications. From the heatmap generated using the negative log p-value of enrichment, we observed shared transcription factors such as FOXB1 and KLF10 across cell types or tissues or epigenetic modifications. Also, some transcription factors are specific to epigenetic modifications. For example, HOXC12 was only enriched in high attribution regions specific to DNA methylation in different cell types.

Furthermore, we identified genes with high attribution regions enriched by MYC binding motifs in the A549 cell line. To validate if MYC indeed binds to these sites, we used the MYC ChIP-seq data of A549 from the ENCODE project (GSM1003607). We found that the MYC ChIP-seq signal peaks on the promoter regions of these genes significantly overlapped with the attribution peaks in the same gene promoters (compared to randomly selected regions, p-value < 0.05). For example, the MYC ChIP-seq signal peak on the NIPSNAP2 promoter region overlapped with the attribution peak of H3K4me3 and Dnase I hypersensitive sites (Fig. 5 B). Similarly, the MYC ChIP-seq signal peak on the GUSB promoter region overlapped with the attribution peaks of H3K4me1 and H3K9me3 (Fig. 5 C). Taken together, we demonstrated that the high attribution regions identified by iSEGnet are potential transcription factor binding regions that may be relevant to gene expression regulation.

### 3.6 Case studies

To further explore the utility of this framework in the context of human disease, we applied iSEGnet on an esophageal cancer dataset [24] and a breast cancer dataset [25] as case studies. The esophageal cancer dataset included DNA methylation and mRNA gene expression profiles available on both cancer and normal tissues (n = 9 in each condition). The breast cancer dataset included H3K4me1, H3K4me3, DNA methylation, and mRNA gene expression profiles on the drug-sensitive breast cancer cell line (MCF7) and the drug-resistant breast cancer cell line (TAMR). There was only one sample available for each epigenetic modification.

**Fig. 6:**
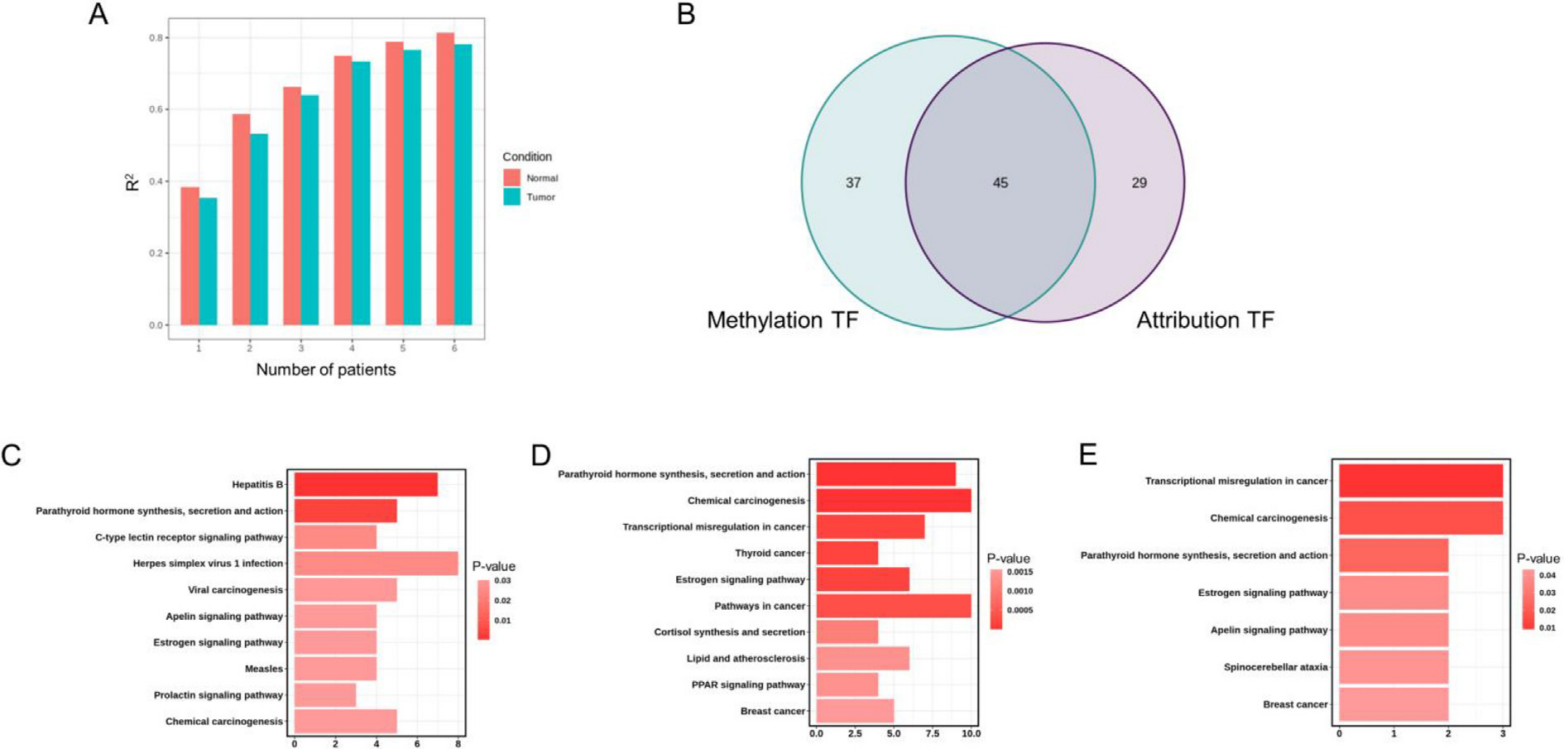
The results of iSEGnet on the esophageal dataset, (A) The R^2^ values of iSEGnet on the esophageal cancer dataset with a varying number of patients as input. (B) The number of transcription factors that are enriched on the regions identified by differentially methylated regions and different attribution regions between cancer and normal tissue. (C) The KEGG pathways enriched for the transcription factors detected from differentially methylated regions. (D) The KEGG pathways enriched for the transcription factors detected from different attribution regions. (E) The overlapped KEGG pathways enriched for the transcription factors identified from differentially methylated regions and different attribution regions.

First, we evaluated whether multiple biological replicates could improve the predictive performance as the esophageal cancer dataset included profiles from nine patients. To test the model performance with multiple replicates, we varied the number of patients in model training. We observed an improved model performance (the R2 increased from 0.38 to 0.82) as more replicates were included, and this effect reached a plateau around n = 5 (Fig. 6 A). This result shows that iSEGnet model performance can benefit from multiple biological replicates.

Next, as the esophageal cancer dataset included tumor and normal tissues, we proceeded to ask the following question: for differentially expressed (DE) genes, can we identify key regulatory regions by comparing attributions derived from the iSEGnet models between tumor and normal tissues? For a given region of a DE gene, if the attribution levels from the two conditions are significantly different, then this region might have regulatory functions that lead to differential expression. We identified the site-specific attributions from both tumor and normal models for every DE gene. We defined a site as a differential attribution site by a threshold on the mean difference of attributions between the two conditions, i.e., a site is a differential attribution site if the difference is among the top 10 % of all the input regions. Then, we retrieved the transcription factor binding motifs on each differential attribution region with FIMO from the MEME suite. The detected motifs belonged to 74 transcription factors. Compared to the transcription factors identified from individual differentially methylated regions with the same approach, 45 of the transcription factors are overlapped (Fig. 6 B). The transcription factors detected on the individual differentially methylated regions are enriched on non-cancer pathways (Fig. 6 C). On the other hand, the KEGG pathways enriched by the 74 transcription factors are cancer-related pathways, such as Transcriptional dysregulation in cancer (hsa05202) and Pathway in cancer (hsa05200) (Fig. 6 D). The overlapped transcription factors between cancer and normal tissue are also cancer-related (Fig. 6 E). These results demonstrate that attribution of the regions derived from iSEGnet could be used to uncover important regions that may be involved in dysregulation of gene expression in human disease.

**Fig. 7:**
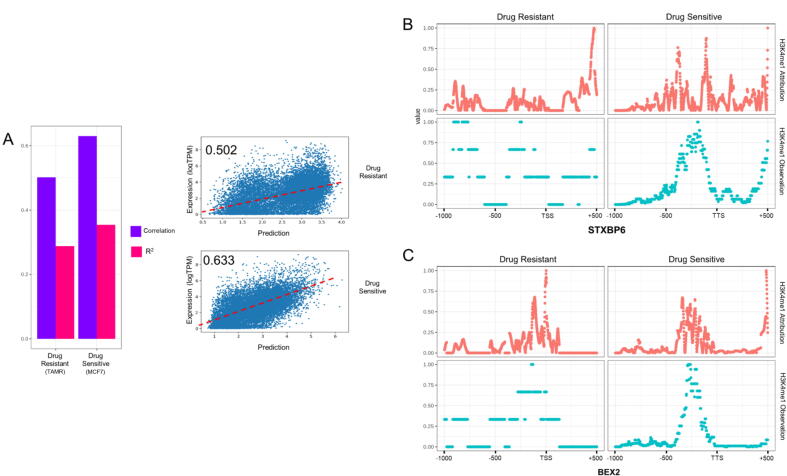
The results of iSEGnet on the breast cancer cell line data (A) The performance of iSEGnet on drug-resistant (TAMR) and drug-sensitive (MCF7) breast cancer cell lines. (B), (C) The observed H3K4me1 ChIP-seq signals and the iSEGnet identified attributions on the promoter region of STXBP6 and BEX2, respectively.

The breast cancer dataset only included one sample for each cell line. The R^2^ values and correlations of iSEGnet were in the range of (0.28, 0.32) and (0.50, 0.63), respectively (Fig. 7 A). We used the same approach to identify the differential attribution regions of the DE genes (determined by fold-change) between the drug-sensitive cell line and drug-resistant cell lines. We found that the transcription factors binding to these regions are enriched for cancer-related KEGG pathways. However, the enriched pathways are not significantly different from those identified from the differential regions of the observed signals (Supplement Fig. S9-S11). Additionally, we found that the observed epigenetic signals are not significantly different at several differential attribution regions. For example, the observed H3K4me1 signals in one DE gene, STXBP6, were high in both drug-sensitive and drug-resistant cell lines at the region 500bs upstream of TSS. However, the attribution of this region was differentially higher in the drug-sensitive cell line (Fig. 7 B). Similarly, for the DE gene, BEX2, there were differentially high attributions at the region 300 bp to 500 bp downstream of the TSS, but the observed H3K4me1 ChIP-seq signals at the same region were not significantly different (Fig. 7 C). These examples suggest that the attribution analysis in iSEGnet may reveal alternative regulatory regions even when observed epigenetic signals do not show a significant difference between the two cell lines.

## 4 Discussion

In this study, we presented iSEGnet, a deep convolutional neural network, which predicts gene expression using epigenetic modifications and DNA sequences of promoter and transcription termination regions. Among various combinations of regions explored, the optimal input regions for iSEGnet, i.e., the combination of [-1000 bp, +500 bp] around TSSs and [-500 bp, +500 bp] around TTSs, generated the best performance. We demonstrated that iSEGnet outperforms other machine learning models, such as support vector machine and random forest, using data from the six cell lines/types obtained from the ENCODE project. Employing the method of Integrated Gradients, we identified the regulatory regions and epigenetic modifications highly relevant to predicting gene expression for individual genes. We further showed that these regions may have regulatory activities by identifying the enrichment of transcription factor binding motifs and overlapping with the peak regions of the corresponding transcription factor ChIP-seq signal. Finally, we applied iSEGnet to two cancer multi-omics datasets to further demonstrate that iSEGnet could be used to identify specific regulatory regions relevant to differential expressed genes between distinct conditions, such as tumor and normal tissues. Thus, iSEGnet is a framework integrating multi-omics of small replicates to discover important transcription factors and regulatory regions that might influence gene expression under different conditions.

iSEGnet is a deep learning architecture with several convolutional and densely connected layers. It learns non-linear mappings from two inputs - epigenetics modifications and DNA sequence - to gene expression. To effectively integrate these two modalities of information, we considered several factors in the architectural design. First, the input data derived from the DNA sequence and the epigenetic input data have different sizes. Namely, the DNA sequence input has four columns, whereas the epigenetic input has multiple columns, depending on the available epigenetic modification data. The proposed model needs the flexibility to integrate these two inputs. Second, since DNA sequence and epigenetic modifications regulate gene expression in an associative way, the deep neural network architecture needs to include epigenetics modification data and the corresponding DNA sequence for each given position in the genome. For the first factor, our model has two key input sources. As shown in Fig. 1, we use two convolutional layers to extract features from a single perspective and make the data from two sources of equal size. For the second factor, we concatenate the outputs from previous convolutional layers by column. This step enables the data integration from the same position to reflect the associative regulation of DNA sequence and epigenetics modifications on gene expression. Thus, the architecture using fully connected layers right after the 2nd convolutional layers cannot maintain the associative information of DNA sequence and epigenetic modification on the same site. This could explain why iSGEnet exhibits better performance as shown in Supplement Figure S2. Another architecture in ExPecto [16] uses a sequential design from DNA sequence to epigenetics factors to predict gene expression values. This type of architecture can reveal the potential regulation pattern from DNA sequence to known epigenetics modification to gene expression. However, ExPecto requires generating 2002 epigenetics features (including histone markers, chromatin accessibility, and TF features) to achieve the best gene expression prediction. On the other hand, iSEGnet can leverage limited epigenetics data typically available in standard biological experiments to generate similar performance. For example, ExPecto reported the correlations between the predicted and observed gene expression in the range of 0.40 to 0.82 in different cell types, whereas iSEGnet models had correlations from 0.68 to 0.89 (Supplement Fig. S4). Thus, ExPector and iSEGnet have comparable performance in terms of prediction accuracy.

Several studies have shown that epigenetic features could be predicted from the DNA sequence [40], [41], [42]. However, the joint impact of DNA sequence and epigenetics features on gene expression was not investigated. To demonstrate that iSEGnet facilitates such analysis, we compared models' predicted gene expression values with different inputs in A549. Particularly, we selected the top 10 % of genes that showed minimal differences between these models for the predicted expression levels. We found that for genes with a higher epigenetic modification level, the epigenetic signals were sufficient to predict gene expression (Supplement Fig. S6). Similarly, we identified a subset of genes whose DNA sequences possess dominant information for predicting gene expression (Supplement Table 4).

As all expressed genes are treated as learning samples, iSEGnet learns the shared transcription regulatory patterns across genes under a particular condition or in a specific cell type from one copy of omics profiles. Although regulatory regions and epigenetic modifications vary between genes, iSEGnet can detect useful patterns for predicting gene expression from multiple hidden layers and kernels in deep CNNs. However, in the current CNN architecture, there is a risk of losing information about the connection between different segments of regulatory regions. This limitation could be removed by employing recurrent neural networks (RNN). RNN has been widely used in modeling sequential data, such as natural language processing [43] and time-series data analysis [44]. The combination of RNN and CNN has been shown as a promising architecture to predict biological events from sequential data such as DNA sequence [45], [46] and DNA methylation [47], [48], [49].

In iSEGnet, we have chosen the input regions for each gene as [-1000 bp, +500 bp] around TSSs and [-500, +500] around TTSs. We determined these optimal lengths by comparing the model performance of multiple combinations of regions around genes. However, distal regulatory regions, such as enhancers located up to 1 Mb away from TSS [50], also regulate gene expression. However, the locations and lengths of distal regulatory regions vary by genes and are often unknown, making it challenging to consider in the current framework. In a recent study [17], long-range enhancer (100 kb away from TSS) and promotor interactions have been explored to predict gene expression based on DNA sequence alone using a self-attention neural network architecture. While it predicts mutation effects in population eQTL studies, this model is not geared towards uncovering the impact of epigenetic modifications on gene expression.

The consequences of epigenetic modifications such as DNA methylation in gene bodies are important in regulating gene expression [51] and thus should be considered in the future. From the integrated gradient analysis of iSEGnet on the epigenetics data, we found that the histone modifications, H3K4me3 and H3K36me3, have higher average importance in the region from TSS to 500 bp downstream, indicating that the starting part of gene bodies also has an impact on gene expression. Thus, It is likely that if we increase the size of the gene bodies, we might observe additional epigenetics-modified regions that are important to predict gene expression. However, the lengths of the gene bodies vary from gene to gene. To meet the requirement of same-length input in the current iSEGnet framework, we would need to add zeros to the genes of shorter length. However, this augmentation could introduce noise into the data. Therefore, we only considered the fixed region around TSS and TTS in the current version.

Identifying the important features from a deep learning model is challenging but essential for knowledge discovery. Multiple methods have been developed to understand feature importance in machine learning and deep learning models, such as SHAP [52] and Integrated Gradients [23]. While the method of Integrated Gradients has been used in several genomics studies to identify regulators of splicing in important genome regions for a distinct purpose [53], the baseline selection of integrated gradients for DNA sequencing and epigenetics modifications remains an open question. In iSEGnet, we used zero as the baseline to identify the feature importance. However, other baseline settings could be explored in the future. For example, the baseline for DNA sequence data could be [0.25, 0.25, 0.25, 0.25], which mimics the distribution of nucleotides on a random DNA sequence. Alternatively, the baseline for DNA methylation could be one instead of zero, to account for maintenance methylation, where the baseline status is a methylated CpG site, as opposed to *de novo* methylation, an unmethylated CpG site is the baseline.

In the case studies of iSEGnet, we demonstrated that our model could locate the high attribution regions of differentially expressed genes. In the esophageal cancer data, the regions identified by differential attributions are more enriched by the binding motifs of transcription factors related to cancer, compared to the regions detected from the differential methylation levels. In contrast, in the breast cancer cell line data, the KEGG pathways enriched for transcription factors obtained from these two approaches are similar. However, the limited number of samples of breast cancer cell line study could potentially explain this finding. The learned attribution of the model correlates with the observation considerably. However, it is hard to reliably detect distinct regulation of gene expression based on only one copy of the multi-omics profile in the closely related cell lines. With more replicates, iSEGnet may perform better as shown in the esophageal cancer data.

Another limitation of iSEGnet is that it focuses on the impact of epigenetic modifications on *cis*-regulatory regions. It is possible and likely that epigenetic modifications in *trans*-regulatory regions can also impact gene expression. Therefore, the future direction of iSEGnet could include designing novel architectures that can take into account epigenetic modifications in *trans*-regulatory regions. However, this would be contingent on the identification of such regions, which still remains a major challenge in biological studies.

In conclusion, iSEGnet is a useful tool to integrate epigenetic modifications and RNA-seq gene expression data to detect plausible regulatory sites in promoter and TTS regions, as well as infer potential transcription factors from a limited number of biological replicates. The results from iSEGnet may facilitate hypothesis generation for interrogating gene regulatory machinery and provide insights into how distinct epigenetic modifications impact gene expression in discrete regulatory regions. The code of iSEGnet is available at github.com/YDaiLab/iSEGnet.

## Declaration of Competing Interest

The authors declare that they have no known competing financial interests or personal relationships that could have appeared to influence the work reported in this paper.
