# Supplementary material for: Assessing comparative importance of DNA sequence and epigenetic modifications on gene expression using a deep convolutional neural network
Source: Comput Struct Biotechnol J. 2022 Jul 13;20:3814–23. doi: 10.1016/j.csbj.2022.07.014 (PMC9307602; doi:10.1016/j.csbj.2022.07.014)

**Supplement Figure S1. The performance of hyperparameter searching**

We performed hyperparameter tunning to determine the optimal set of hyper-parameters (number of layers, number of kernels, kernel size, λ and dropout rate). We allowed for two convolutional layers in each of the convolutional networks. The kernel size varied from the combination of 16, 32, 64, and 128 with different sizes: 20x7, 50x7, and 100x7. The L2 regularization parameter (λ) in the loss function was selected from 5 different values: 0.0001, 0.01, 0.1, and 1.0. Dropout was selected from 0.1, 0.3, and 0.5. **(A)** The performance of different hyperparameters on A549. **(B)** The performance of iSEGnet on 5 cell types (H3pG2, K562, Large intestine, pancreas, small intestine) with different kernel numbers with L2 regularization parameter (λ) as 0.0001and dropout rate as 0.5. **(C)** The performance of iSEGnet on 5 cell types (H3pG2, K562, Large intestine, pancreas, small intestine) with different L2 regularization parameter (λ) with kernel numbers as 64x128 and dropout rate as 0.5.


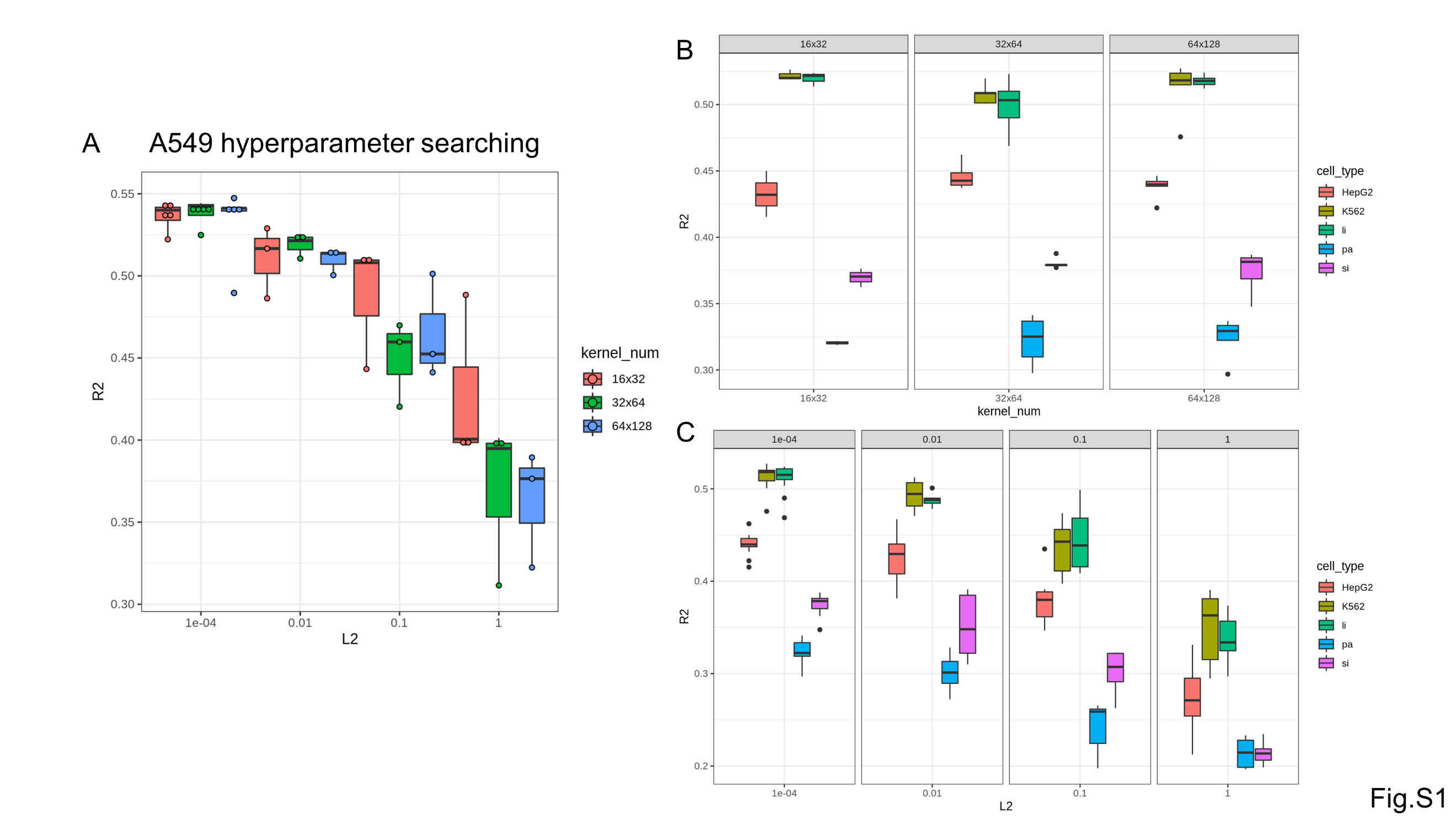


**Supplement Figure S2. The performance of iSEGnet and the other architecture.**

**(A) (B)** The R^2^ values and Pearson’s correlations of iSEGnet and the other architecture, respectively. In the other architecture instead of using a convolutional layer to extract information from the concatenation layer, the fully connected layers right after the concatenation layer was used.


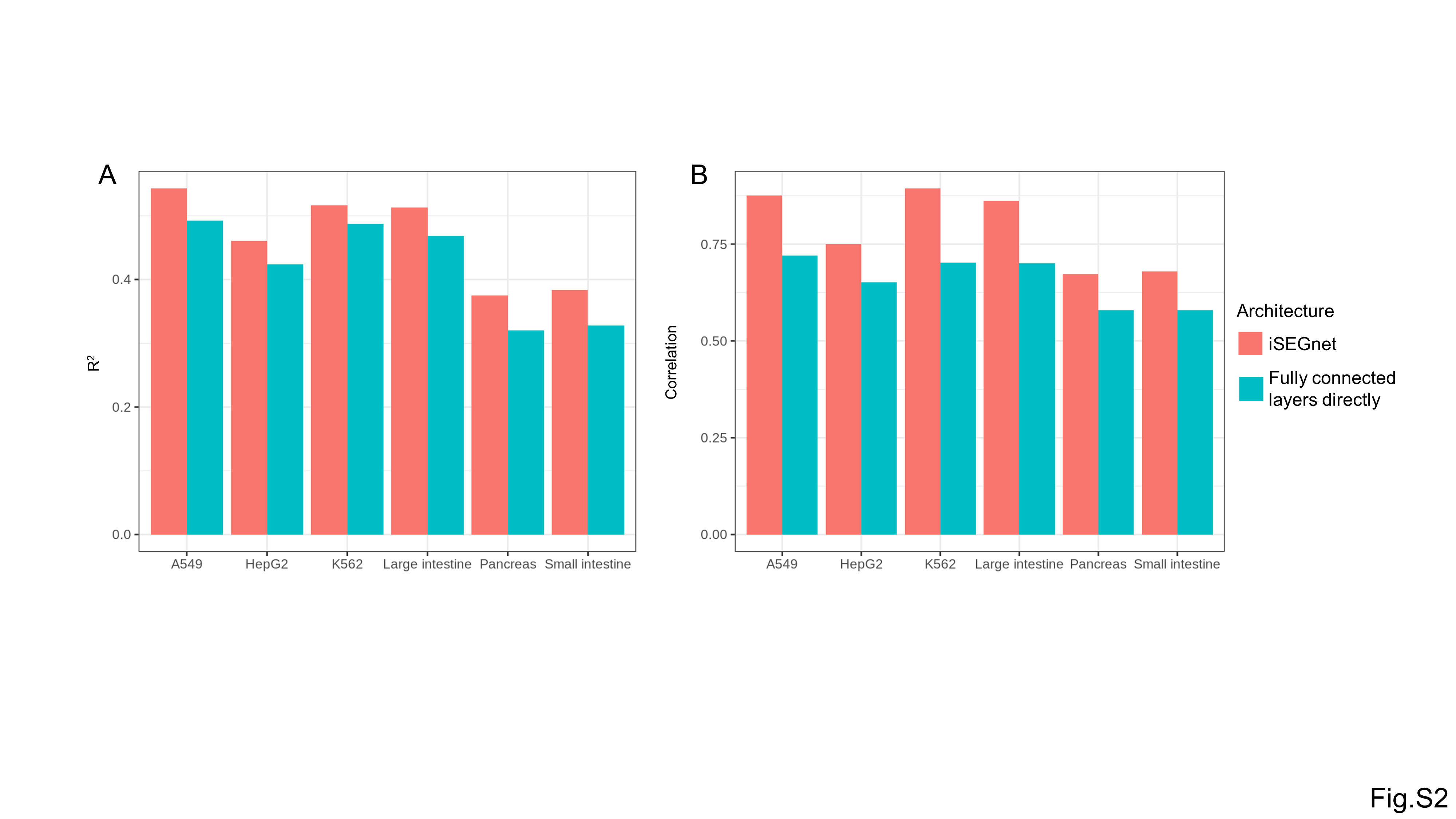

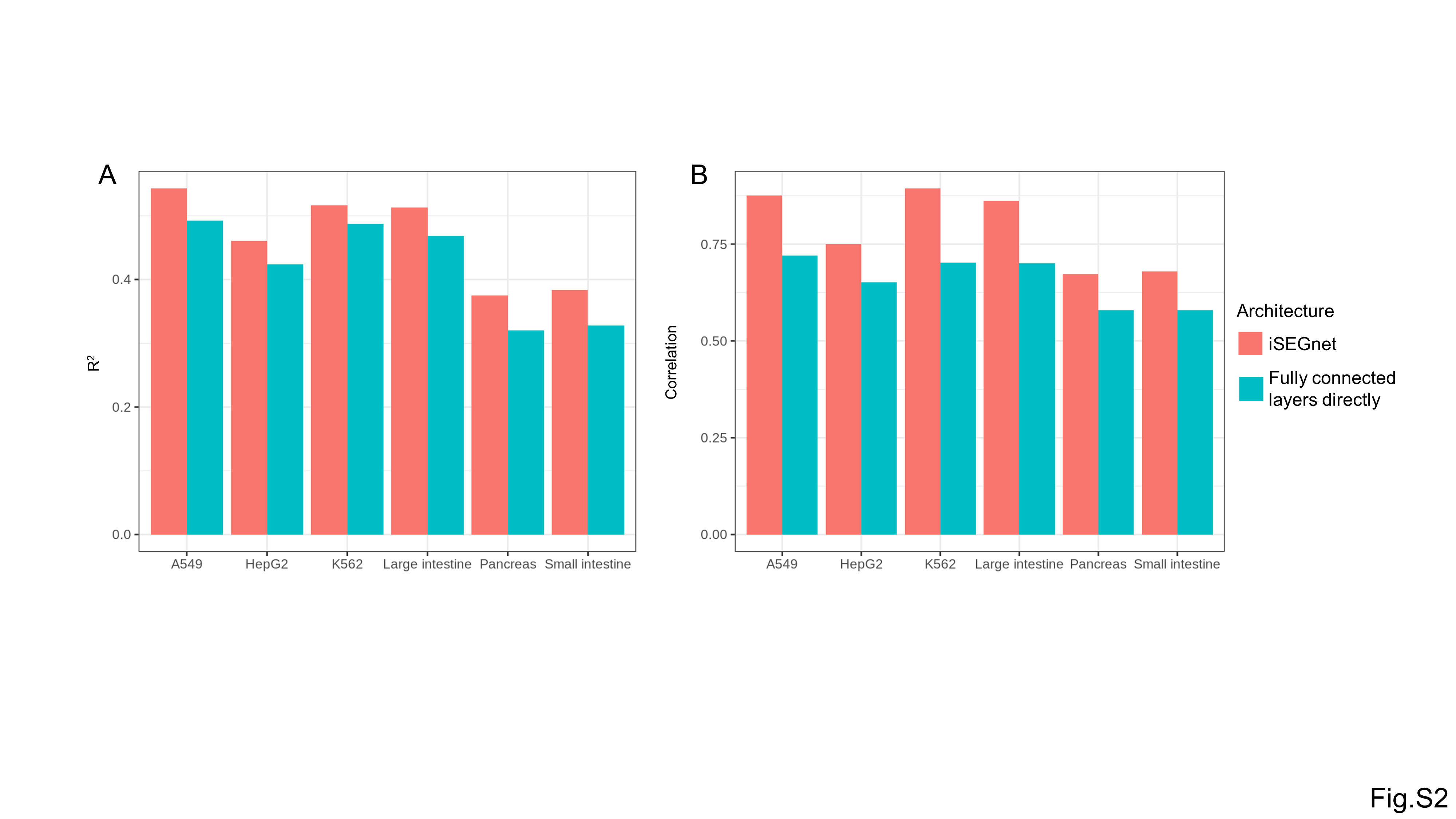


**Supplement Figure S3. The R^2^  values of different numbers of decision trees in random forest models**


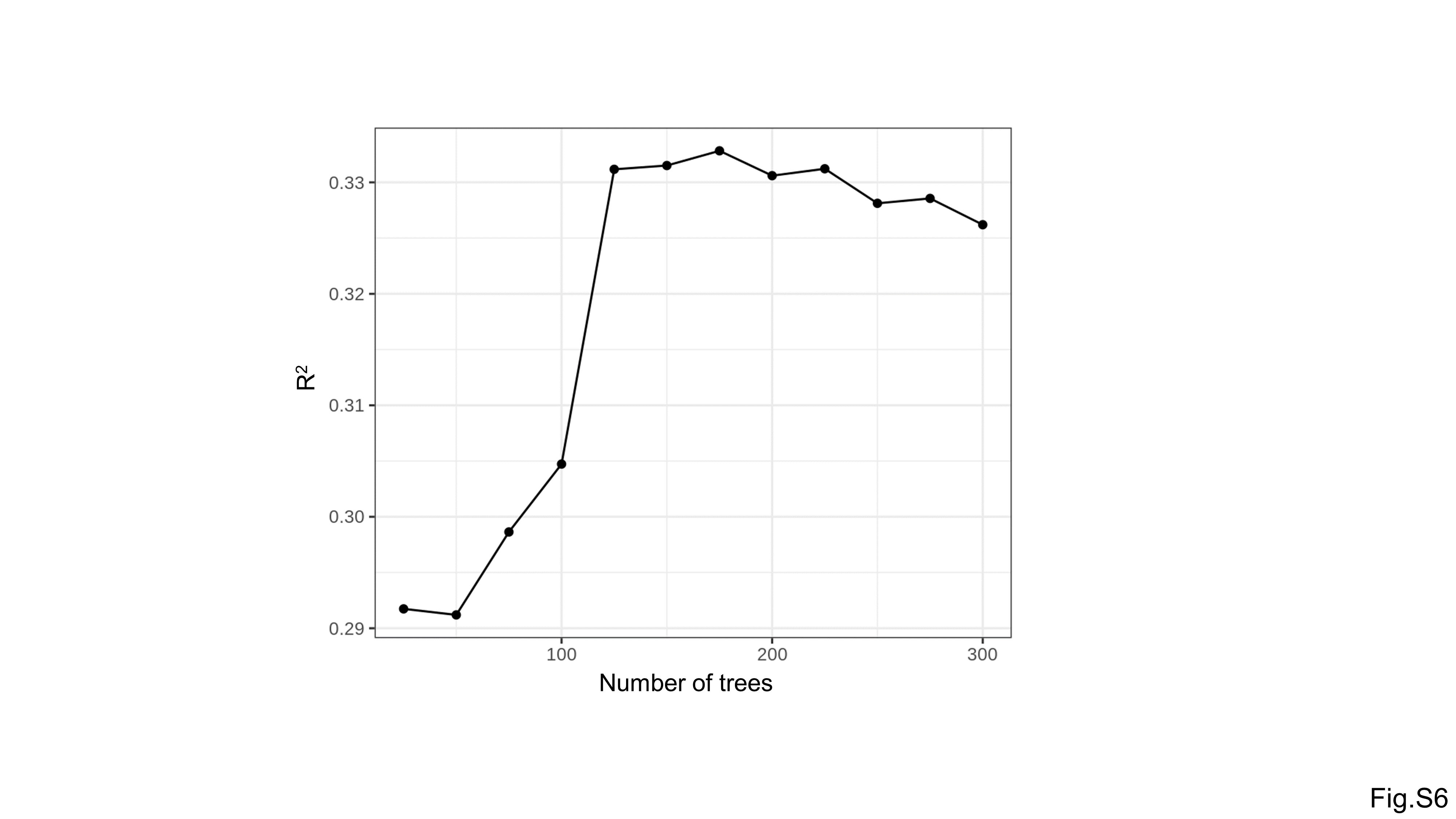


**Supplement Figure S4. The performance of iSEGnet on different cell lines and cell types.**

R^2^ is the coefficient of determination. 𝑟 is the Pearson's correlation.


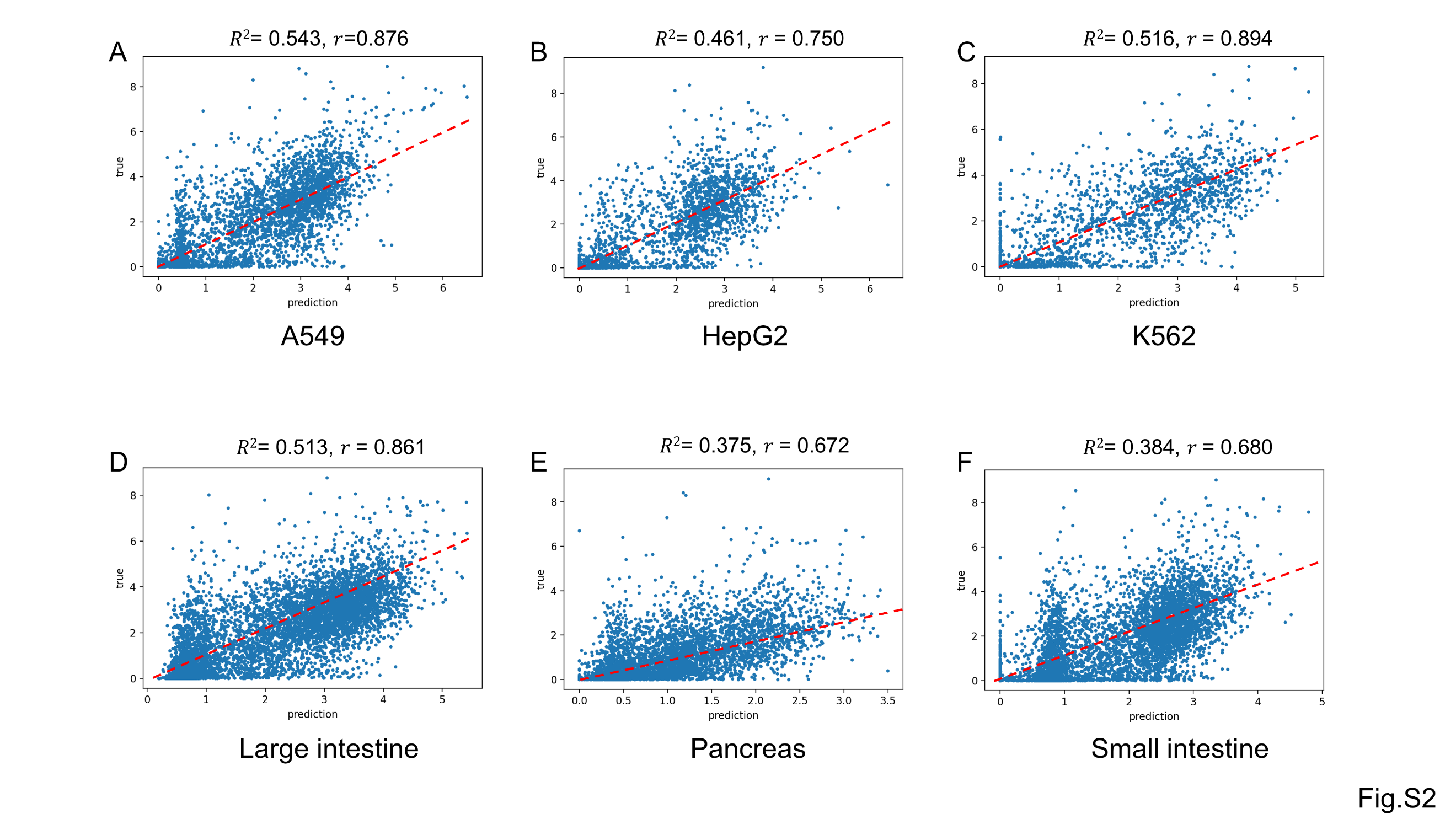


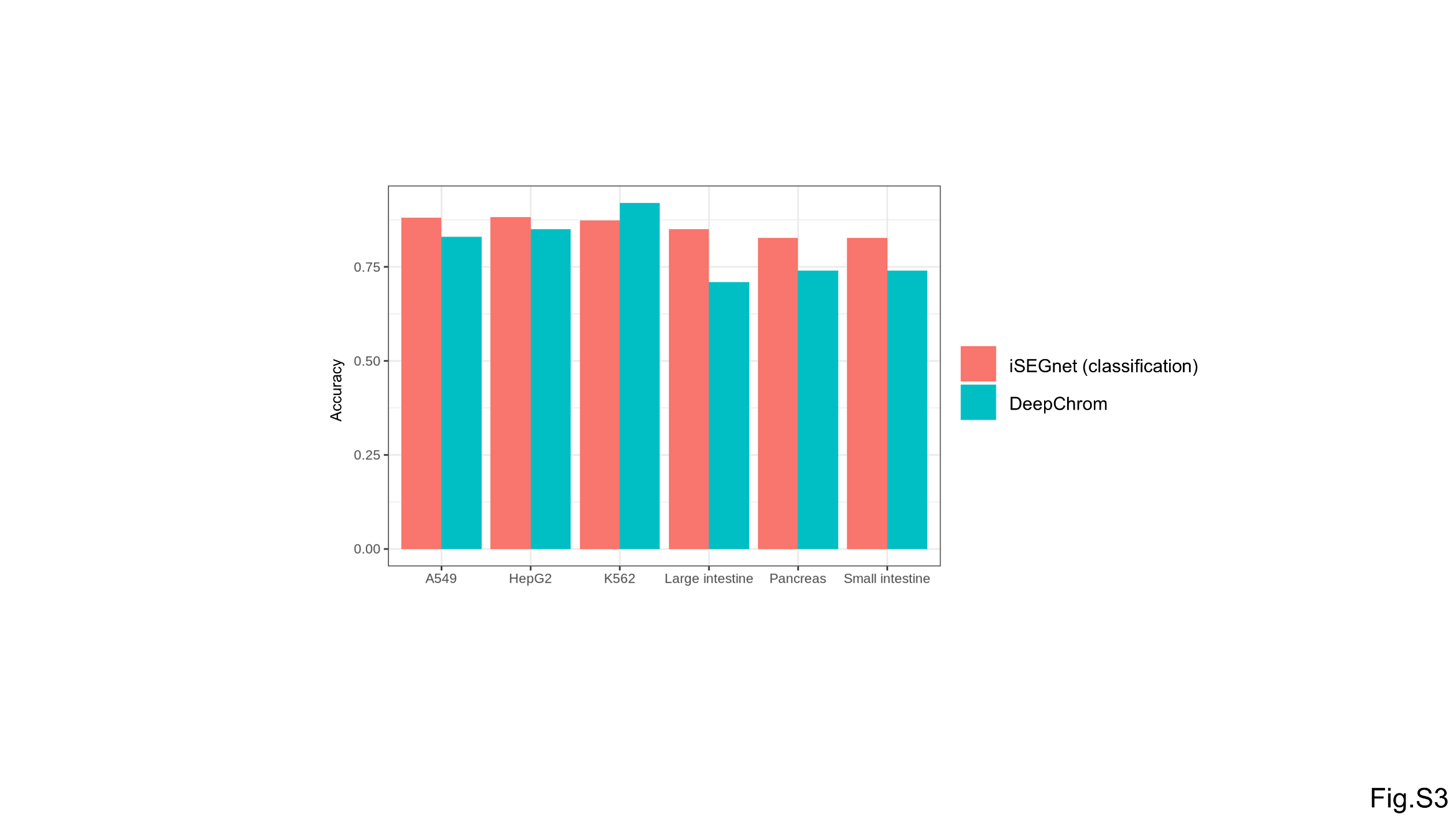


**Supplement Figure S5. The performance of the binary version of iSEGnet and DeepChrom.**


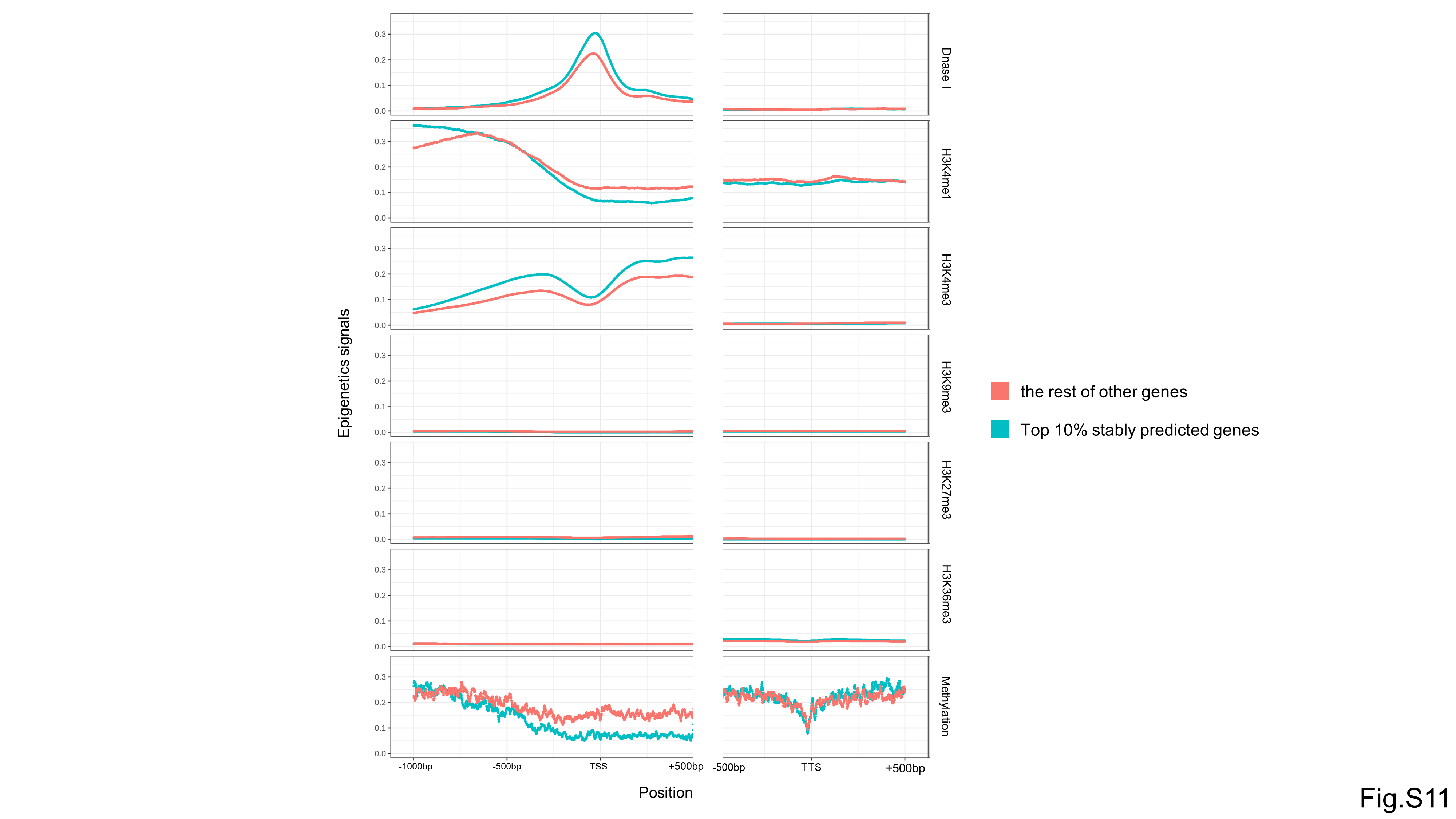


**Supplement Figure S6. The observed epigenetics signals of the top 10% stably predicted genes and the rest of the other genes in data from A594.**

The stably predicted means that the predictions of gene expression remain similar with or without DNA sequcens information.

**Figure S7. The attributions of epigenetic modifications on gene expression by integrated gradients.**

**(A), (B)** and **(C)** The mean predicted site-specific attributions for each epigenetic modification on gene expression across all genes in cell types, large intestine, pancreas and small intestine, respectively.


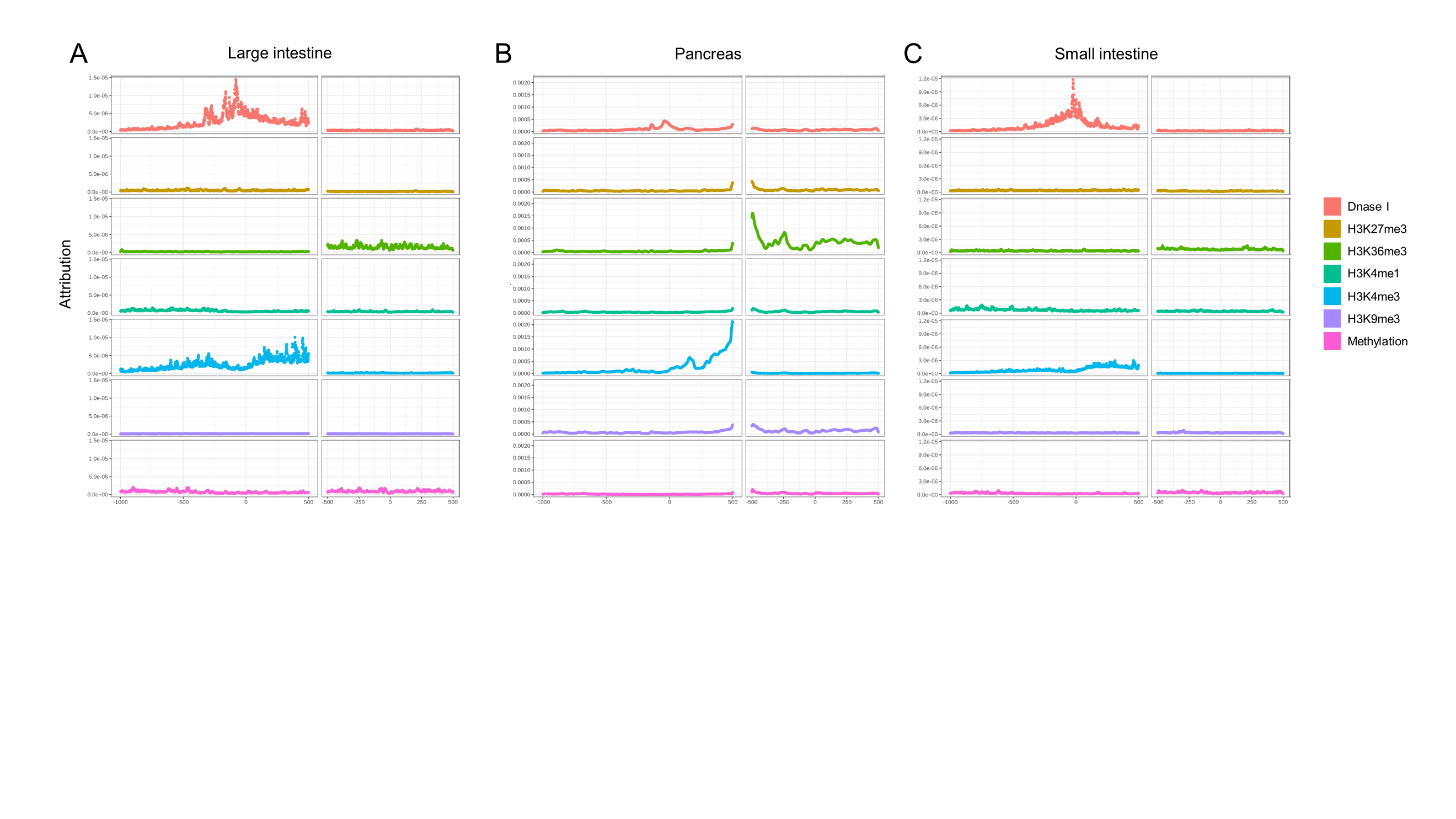


**Supplement Figure S8. The attributions of DNase I hypersensitive site and H3K4me3 for MYC expression.**

**(A)** Attribution of DNase I hypersensitive site for gene expression retrieved by integrated gradients.

**(B)** Experimentally observed of DNase I hypersensitive site for gene expression retrieved by integrated gradient.

**(C)** Attribution of H3K4me3 for gene expression retrieved by integrated gradients.

**(D)** Experimentally observed of H3K4me3 for gene expression retrieved by integrated gradients.


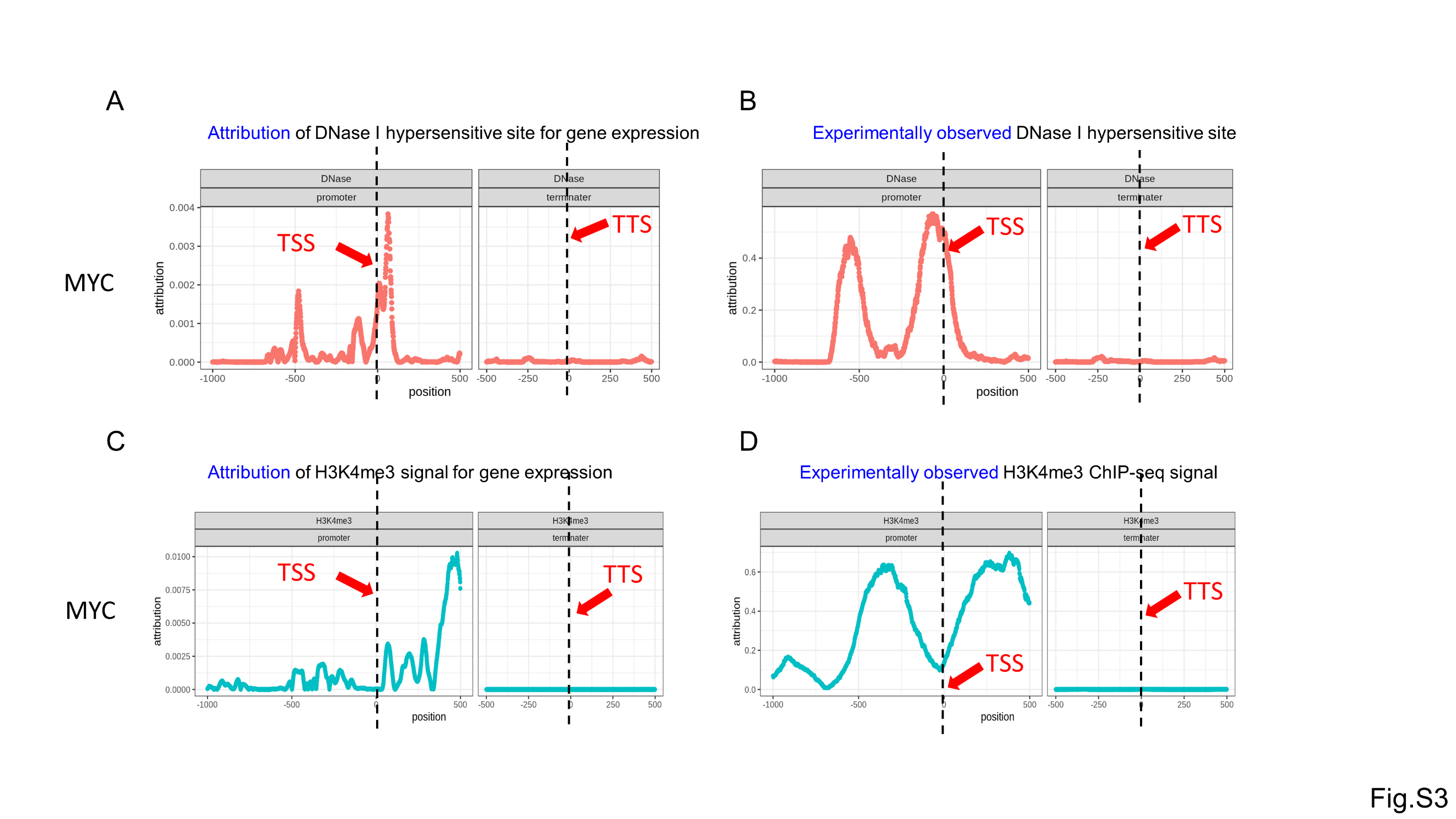


**Supplement Figure S9. The transcription factors that retrieved from H3K4me1 attribution and observed signal on the two breast cancer cell lines.**

**(A)** The number of transcription factors that are enriched on the regions identified by differentially H3K4me1 regions and different attribution regions between drug resistant and sensitive cell lines. **(B)** The KEGG pathways enriched for the transcription factors detected from differentially H3K4me1 regions. **(C)** The KEGG pathways enriched for the transcription factors detected from different attribution regions.


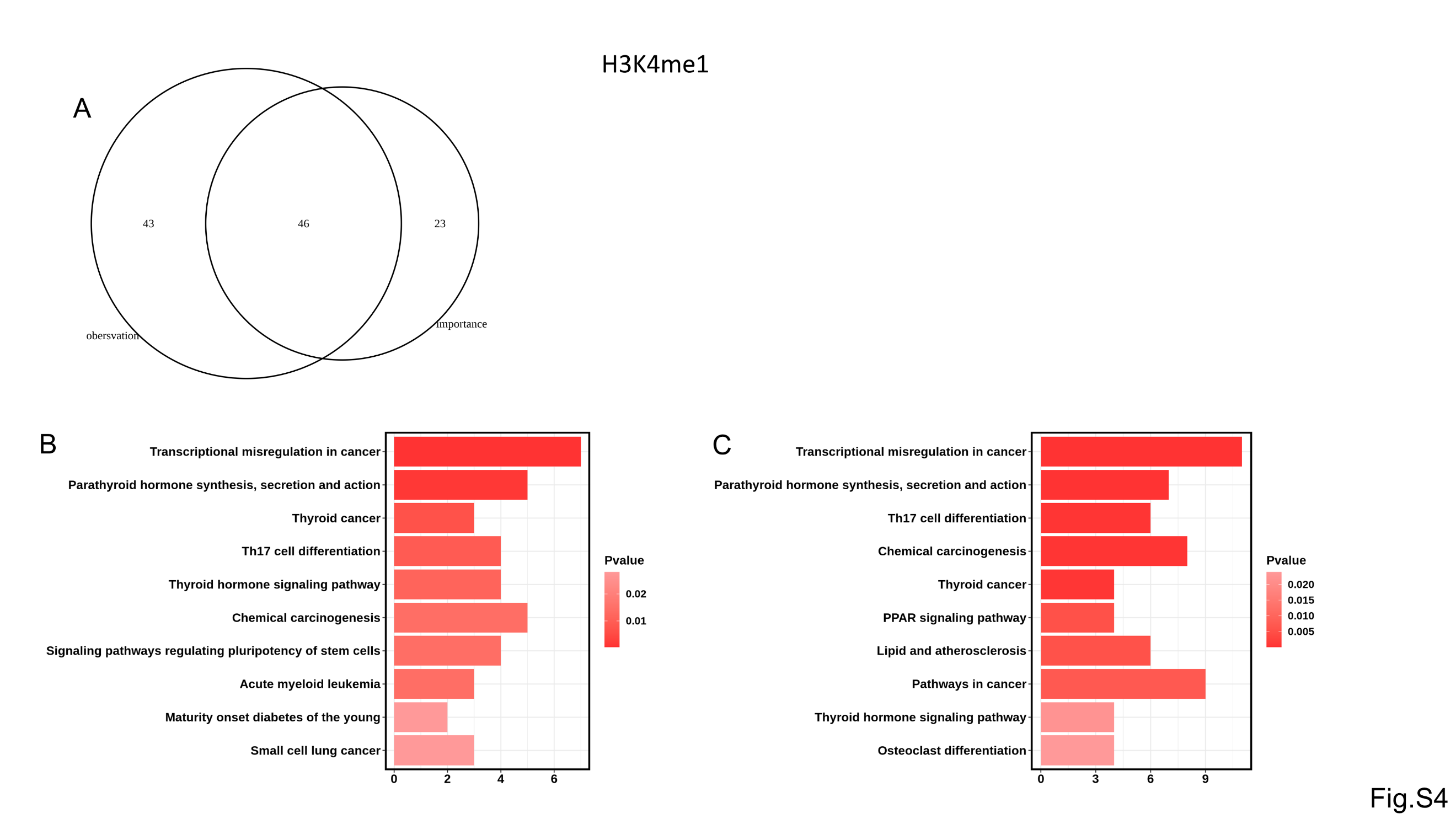


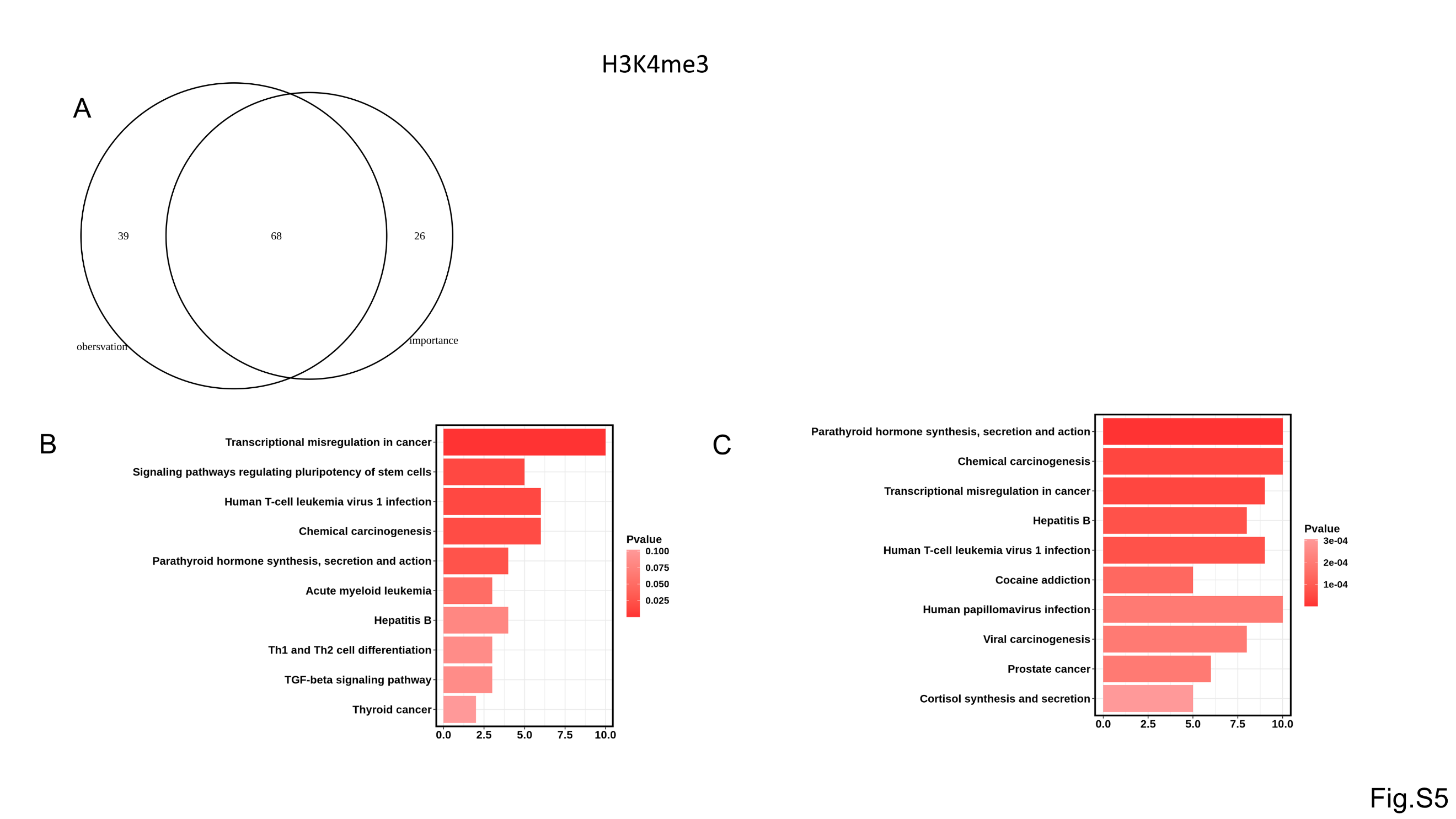


**Supplement Figure S10. The transcription factors that retrieved from H3K4me3 attribution and observed signal on the two breast cancer cell lines.**

**(A)** The number of transcription factors that are enriched on the regions identified by differentially H3K4me3 regions and different attribution regions between drug resistant and sensitive cell lines. **(B)** The KEGG pathways enriched for the transcription factors detected from differentially H3K4me3 regions. **(C)** The KEGG pathways enriched for the transcription factors detected from different attribution regions.

**Supplement Figure S11. The transcription factors that retrieved from methylation attribution and observed signal on the two breast cancer cell lines.**

**(A)** The number of transcription factors that are enriched on the regions identified by differentially methylated regions and different attribution regions between drug resistant and sensitive cell lines.  **(B)** The KEGG pathways enriched for the transcription factors detected from differentially methylated regions. **(C)** The KEGG pathways enriched for the transcription factors detected from different attribution regions.


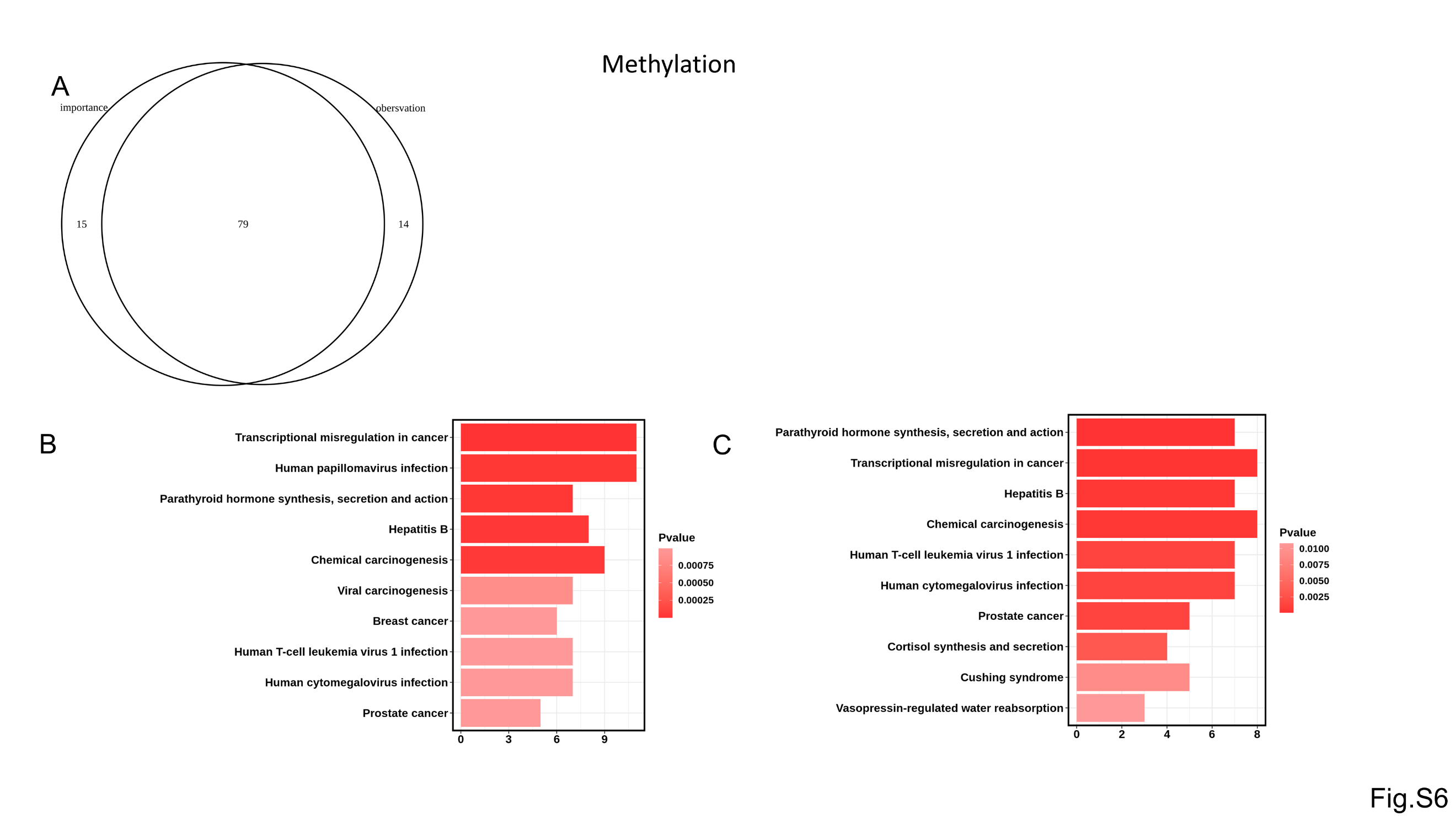

Supplement: Supplementary data 1 [file mmc1.docx]
